# Introduction to Large Language Models (LLMs) for dementia care and research

**DOI:** 10.3389/frdem.2024.1385303

**Published:** 2024-05-14

**Authors:** Matthias S. Treder, Sojin Lee, Kamen A. Tsvetanov

**Affiliations:** ^1^School of Computer Science & Informatics, Cardiff University, Cardiff, United Kingdom; ^2^Olive AI Limited, London, United Kingdom; ^3^Department of Clinical Neurosciences, University of Cambridge, Cambridge, United Kingdom; ^4^Department of Psychology, University of Cambridge, Cambridge, United Kingdom

**Keywords:** dementia, Large Language Model (LLM), Artificial Intelligence, Alzheimer's disease, care, natural language processing

## Abstract

**Introduction:**

Dementia is a progressive neurodegenerative disorder that affects cognitive abilities including memory, reasoning, and communication skills, leading to gradual decline in daily activities and social engagement. In light of the recent advent of Large Language Models (LLMs) such as ChatGPT, this paper aims to thoroughly analyse their potential applications and usefulness in dementia care and research.

**Method:**

To this end, we offer an introduction into LLMs, outlining the key features, capabilities, limitations, potential risks, and practical considerations for deployment as easy-to-use software (e.g., smartphone apps). We then explore various domains related to dementia, identifying opportunities for LLMs to enhance understanding, diagnostics, and treatment, with a broader emphasis on improving patient care. For each domain, the specific contributions of LLMs are examined, such as their ability to engage users in meaningful conversations, deliver personalized support, and offer cognitive enrichment. Potential benefits encompass improved social interaction, enhanced cognitive functioning, increased emotional well-being, and reduced caregiver burden. The deployment of LLMs in caregiving frameworks also raises a number of concerns and considerations. These include privacy and safety concerns, the need for empirical validation, user-centered design, adaptation to the user's unique needs, and the integration of multimodal inputs to create more immersive and personalized experiences. Additionally, ethical guidelines and privacy protocols must be established to ensure responsible and ethical deployment of LLMs.

**Results:**

We report the results on a questionnaire filled in by people with dementia (PwD) and their supporters wherein we surveyed the usefulness of different application scenarios of LLMs as well as the features that LLM-powered apps should have. Both PwD and supporters were largely positive regarding the prospect of LLMs in care, although concerns were raised regarding bias, data privacy and transparency.

**Discussion:**

Overall, this review corroborates the promising utilization of LLMs to positively impact dementia care by boosting cognitive abilities, enriching social interaction, and supporting caregivers. The findings underscore the importance of further research and development in this field to fully harness the benefits of LLMs and maximize their potential for improving the lives of individuals living with dementia.

## Introduction

As the global population ages, dementia emerges as one of the most pressing and multifaceted healthcare challenges (Parra et al., 2019). More than 55 million individuals worldwide are currently living with dementia, with over 60% of these cases occurring in low- and middle-income countries. Furthermore, approximately 10 million new cases of dementia are diagnosed annually (WHO, 2023). Characterized by progressive cognitive decline that impedes daily functioning, dementia not only impacts the affected individuals, but also their caregivers, families, and the healthcare system at large. Furthermore, dementia is frequently diagnosed late or misdiagnosed (Fischer et al., 2017), while the limited availability of caregiver support post-diagnosis compounds the challenges faced by all involved. It becomes imperative for dementia care and research to develop innovative solutions for improved diagnosis, effective treatment and caregiving, ultimately reducing the global burden of this condition.

Amidst this backdrop, the rise of advanced computational tools and Artificial Intelligence (AI) technologies offers a beacon of hope. A branch of AI known as Large Language Models (LLMs), with their capacity to understand, generate, and interact using natural language, are at the forefront of these technological innovations (Bubeck et al., 2023; Huang and Chang, 2023; Khurana et al., 2023; Min et al., 2023). In the realm of dementia care and research, LLMs present unique opportunities to revolutionize diagnostic strategies, therapeutic interventions, and patient-caregiver communication. Yet, for all their promise, LLMs also bring forth a range of ethical, practical, and scientific challenges (Blodgett et al., 2020; Gabriel, 2020; Liao, 2020; Dobbe et al., 2021; Barocas et al., 2023; Floridi and Floridi, 2023; Gallegos et al., 2023; Kasneci et al., 2023; Li and Zhang, 2023; Wang et al., 2023; Bzdok et al., 2024). This paper aims to elucidate the prospects and potential pitfalls of employing LLMs in the domain of dementia care and research, paving the way for informed and judicious integration of these powerful tools in real-world settings.

Our key contributions are as follows:

1. To our knowledge, this is the first publication specifically reviewing LLMs in the context of dementia management and care. Previous reviews surveyed AI in dementia more broadly (de la Fuente Garcia et al., 2020; Lee et al., 2021; Richardson et al., 2022; Borchert et al., 2023; Tsoi et al., 2023) or focused on AI for prediction and early diagnosis (Stamate et al., 2020; Li et al., 2022; Merkin et al., 2022; Borchert et al., 2023).
2. We propose and thoroughly discuss several application scenarios where LLMs can be useful to people with dementia, including navigation aid, reading/writing assistance, and conversational services.
3. We present the results of a survey of people with dementia (PwD) and supporters wherein we investigated their experience with AI and LLMs, their evaluation on the usefulness of the presented application scenarios, and their priorities that AI software developers should consider (e.g., privacy, ease of use).

In the next section, we briefly review the dementia literature, before introducing the application of LLMs in this field.

### Dementia overview

A detailed introduction into dementia, its epidemiology, various subtypes and diagnosis, risk factors, and treatment is included in the Supplementary material A. For brevity, we only provide a summary here. Dementia is a major public health priority (Prince et al., 2015), with the number of affected individuals expected to triple by 2050 (Nichols et al., 2022), creating significant economic and social challenges (Nandi et al., 2022). It encompasses various brain disorders characterized by a decline in cognitive and motor functions due to brain cell loss. Common types include Alzheimer's disease, vascular dementia, dementia with Lewy bodies, and frontotemporal dementia, each associated with specific brain regions and symptoms. Mixed dementia involves concurrent brain changes from multiple dementia types (Schneider et al., 2007; Kapasi et al., 2017).

Alzheimer's disease, the most prevalent cause of dementia, involves memory lapses, word-finding difficulties, and mood swings, with damage often starting in the hippocampus (Sheehan, 2012; Jack et al., 2018; Lane et al., 2018; Armstrong et al., 2024). Most Alzheimer's cases are sporadic with late onset, but a rare early-onset form typically appears before the age of 65 (2023 Alzheimer's Disease Facts and Figures, 2023). Vascular dementia arises from damage to the brain's blood vessels and is associated with cognitive impairments such as impaired judgment, planning difficulties, and mood fluctuations (Iadecola et al., 2019; Bir et al., 2021). Dementia with Lewy Bodies features abnormal Lewy body protein deposits in the brain. It manifests as visual hallucinations and Parkinson's-like movement problems, often coexisting with Alzheimer's pathology (Kane et al., 2018). Frontotemporal Dementia often affects younger adults (45–60 years) and impacting cognition, personality, and behavior with various subtypes based on specific symptoms and pathologies (Coyle-Gilchrist et al., 2016; Olney et al., 2017; Raffaele et al., 2019; Murley et al., 2020).

Primary risk factors include age, genetics, and family history (2023 Alzheimer's Disease Facts and Figures, 2023). However, modifiable risk factors such as cardiovascular health and lifestyle choices can significantly impact dementia risk (Livingston et al., 2020). Current treatments focus on symptom management with emerging pharmacological advancements aimed at altering disease progression. Non-pharmacological interventions and comprehensive care strategies are vital for enhancing quality of life. Moreover, proactive management involves care strategies, including treatment optimization, caregiver training, and community support networks, to improve patient outcomes and enhance caregiver wellbeing.

As reviewed below, the use of AI technology for dementia management and care offer promising avenues for personalized treatment and continuous monitoring of disease progression. Traditional pharmacological treatments, lifestyle interventions and AI technology can work together in a comprehensive approach to address the multifaceted challenges of this complex neurological condition. By combining these different methods, we may be able to improve outcomes for patients with dementia, alleviate caregiver burden, and better meet the needs presented by dementia.

### Artificial Intelligence for dementia

Artificial Intelligence (AI) applications in Alzheimer's Disease initially focused on neuroimaging, particularly tracking brain volume changes to identify brain atrophy (Giorgio et al., 2020; Brierley, 2021; Lombardi et al., 2022; Qiu et al., 2022; Borchert et al., 2023). Early examples include an AI algorithm achieving 92.36% accuracy in classifying Alzheimer's Disease based on Magnetic Resonance Imaging scans (Zhang et al., 2015) and another predicting Alzheimer's Disease over 75 months earlier with 82% specificity and 100% sensitivity (Ding et al., 2019). Beyond neuroimaging, AI research aims to make cognitive tests (Li et al., 2022), speech assessments (O'Malley et al., 2020), and dementia screenings reproducible on a larger scale, enhancing accessibility, even in remote populations. A Canadian medical imaging company has developed a technology utilizing retina scans to detect amyloid buildup, a protein associated with Alzheimer's Disease in its early stages (Dangerfield and Katherine, 2023).

As a special instantiation of AI, Large Language Models (LLMs) have been only scarcely explored in the context of dementia care and management. In the Method section, we introduce LLMs, their general architecture, training and limitations and risks associated with LLMs. We then revisit these topics in the context of dementia. Finally, we introduce a questionnaire what was sent out to people with dementia (PwD) and supporters (e.g., caregivers, family members, or nurses). We investigated their views on various application scenarios as well as their priorities for LLM-powered digital apps (e.g., ease of use, data privacy).

## Method

### Large Language Models (LLMs)

The years 2023–2024 have been a period of tremendous growth for LLMs both in terms of computational capability and public exposure. In January 2023, OpenAI's language model known as ChatGPT reached the 100 million users mark 2 months after its release, making it the fastest growing consumer app to date (Hu, 2023). Spurred by the stellar success of OpenAI, big tech competitors Google and Meta soon followed suit, releasing new versions of their respective competitor models PaLM2 (Ghahramani, 2023; Mauran, 2023), Bard (Hsiao, 2023) and Llama (Touvron et al., 2023). In this section, we review the technological fundamentals of LLMs and the way they are trained, finetuned and deployed, their risks and limitations, and we review some state of the art models. We keep the technical discussion at a conceptual level in order to make it useful to a broad audience. Table 1 provides a glossary with a concise description of some of the technical terms used in the next subsections. A brief overview of the history of LLMs is provided in the Supplementary material B.

**Table 1:** Glossary of terms relevant in the context of Large Language Models.

**Term**	**Definition**
Alignment	Process of ensuring the model aligns with human values, ethical guidelines, and intended uses, while minimizing harmful outputs and biases (see Section *Bias and alignment*).
Artificial Intelligence (AI)	Algorithms that can perform tasks typically requiring human intelligence, such as problem-solving, learning, perception, and decision-making. Typically, AI systems excel only at a single task, i.e. do not generalize/transfer across a range of tasks/problems.
Artificial General Intelligence (AGI)	An emerging form of AI that possesses the capacity to understand, learn, and apply its intelligence across a wide range of tasks at a level comparable to or exceeding human capability. AGI models excel at a large number of tasks simultaneously (see Section *Artificial General Intelligence and psychology*).
Bias	Skewed or unfair tendencies and associations present in the model's responses, often as a result of imbalances or prejudices within the training data (see Section *Bias and alignment*).
Context window	The maximum amount of text the model can process at once, setting a limit on the amount of information it can use when generating responses.
Finetuning	Further refinement of a pretrained model on a specific, often smaller dataset, to adapt and enhance its performance for particular tasks or subject areas. The finetuning stage is essential for turning the model into a helpful assistant (see Section *Training*).
Hallucinations	Factually incorrect, nonsensical, or irrelevant information produced by the model that is not supported by the input data or real-world facts, often as a result of misinterpreting the context or overgeneralizing from its training.
In-context learning	The model's ability to understand and respond appropriately based on the immediate context or examples provided within a given input, without additional external training or finetuning (see Section *Training*).
Machine Learning	A subset of artificial intelligence that involves the development of algorithms and statistical models that enable computers to improve their performance on a specific task through learning from data and experience.
Overreliance	The tendency to excessively depend on the model's outputs without thorough critical evaluation, potentially leading to unwarranted trust in inaccurate, biased, or inappropriate responses generated by the model.
Pretraining	Initial phase of training where the model learns general language patterns and understanding from a vast, diverse dataset, before being finetuned on specific tasks or domains.
Prompt	User input or instruction given to the model, which guides and influences its subsequent text generation or response.
Prompt engineering	Skillful crafting and optimization of prompts to effectively guide and improve the model's responses, ensuring more accurate, relevant, or creative outputs.
Token	Basic unit of text, such as a word, part of a word, or punctuation, used for processing and generating language.
Training	Adjusting the *weights* (parameters in a model) to accurately interpret and generate language based on the patterns learned from its training data. Training involves multiple stages, namely pretraining, finetuning, and sometimes in-context learning (see Section *Training*).
Transformer	The currently dominant model architecture for language models. It efficiently processes text using mechanisms like attention to capture dependencies and relationships between words (Vaswani et al., 2017).
Weights	The parameters within a model that determine how it interprets and generates text. The number of these parameters is usually in the billions.

#### Using Large Language Models

**Figure 1:**
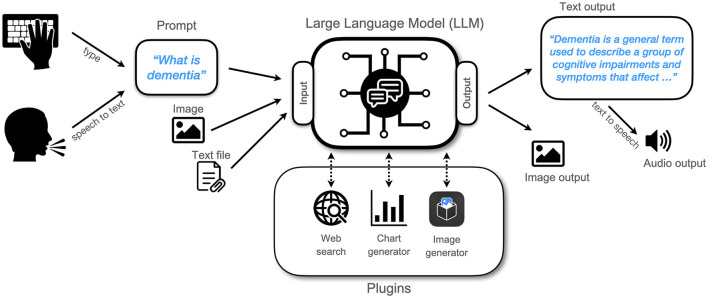
Flowchart showing how a user interacts with a Large Language Model.

Figure 1 summarizes the interaction of a user with an LLM. Users can typically type input prompts using a browser window with a chat interface. Additionally, many models provide an Application Programming Interface (API) that allows for computer programs or smartphone apps to access an LLM in the background. Most LLMs cannot be efficiently deployed on a local device because of their enormous requirements in terms of processing power and memory. Therefore, in many cases the LLM will be running in a data center and accessed via an internet connection. The user provides a prompt by either typing it in directly or using speech that is then converted to text using a separate speech-to-text algorithm. The prompt can be a question (“What is dementia?”), a statement (“I am happy today”), or a set of instructions (“Generate a point-by-point list of activities to do in London today, taking into account the current weather. For lunch, suggest good vegetarian restaurants around Greenwich.”). Auxiliary data such as images or text files can be provided and the text prompt can include a reference to the data (“Describe the image”). During the processing of the prompt, some LLMs can recruit software plugins such as web search to fetch news items, or chart and image generators to create visuals. The LLM autonomously generates control commands to operate the plugins and it incorporates their output. The LLM then returns text output to the user, which can be converted to audio using a text-to-speech algorithm. Alternatively, outputs can take the form of other modalities such as images.

The quality of the returned text can often be improved by carefully crafting the prompts given to the model. This is known as *prompt engineering*. A few such techniques have been developed and have shown to lead to higher accuracy and better responses. *Chain-of-thought* prompting involves giving structured, multi-step instructions or explanations within the prompt, guiding it to generate step-by-step reasoning in its responses, akin to a human solving a complex problem (Wei J. et al., 2023). *Tree-of-thoughts* expands on this idea by encouraging the model to explore multiple possible lines of reasoning simultaneously, akin to a branching tree of ideas (Yao et al., 2023). In analogical prompting, the model is prompted to recall examples relevant to a new task and then afterwards solve the initial problem (Yasunaga et al., 2023).

#### Training

**Figure 2:**
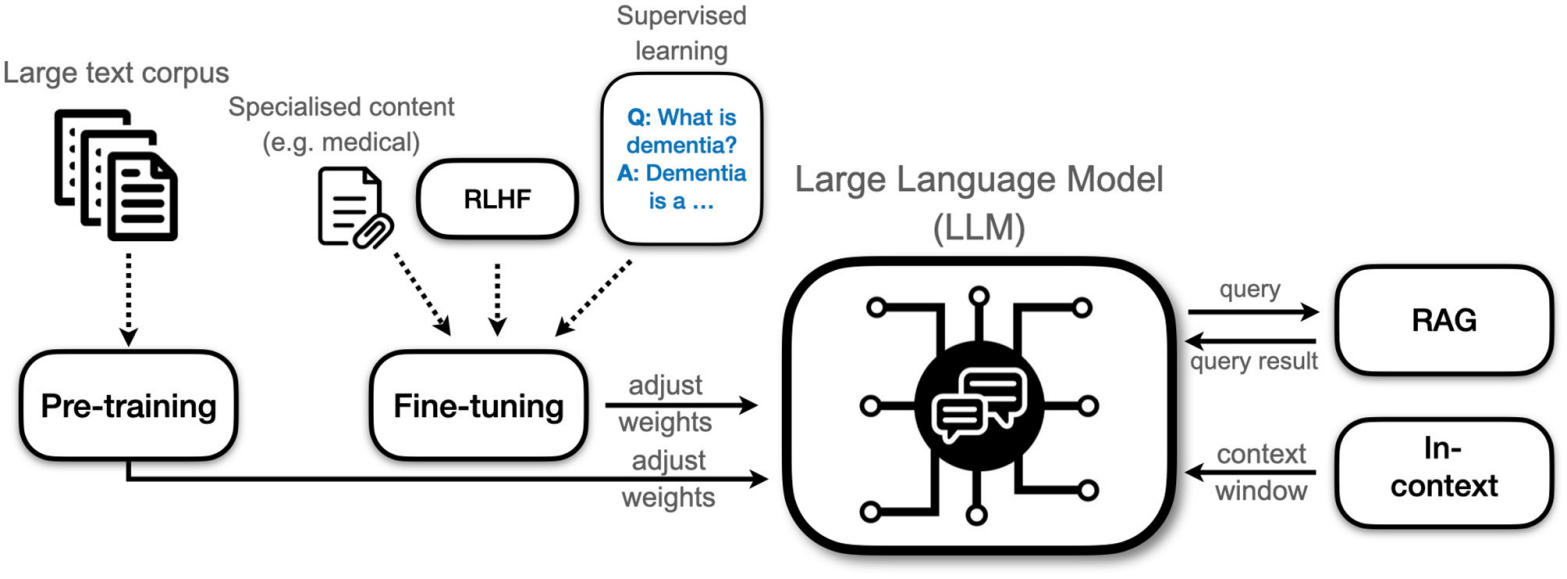
Different types of training an LLM. Pre-training and fine-tuning involves adjusting the weights of the model whereas in-context learning and retrieval-augmented generation (RAG) works for deployed models and does not change the internal structure of the model. RLHF, Reinforcement learning with human feedback.

In this section we will explain the basic principles of how LLMs are trained from scratch. Most models are based on the transformer architecture that was introduced by Vaswani et al. (2017). Training involves changing the weights of the model. Weights determine how it interprets and generates text. Their number is usually in the billions. Weights form the parameters that encode the model's understanding of language and its knowledge about the world. Note that training a model is something most users will never do themselves. Training a state of the art model requires prohibitively large resources of data and compute power, so it is something mostly done by large tech firms and well-funded startups. Training typically progresses through two stages: pretraining and finetuning. An additional in-context learning stage can happen during the interaction with the user, allowing further adaptation. Figure 2 depicts the different phases of training.

##### Pretraining

In the pretraining stage, the model trains on a large text corpus using unsupervised objectives. The objective is to teach the model to understand general linguistic patterns and structures, and to encode world knowledge and facts in its weights. For instance, it learns that “Albert Einstein” was a physicist and Nobel prize laureate, or that London is the capital of the United Kingdom. It can be conceived of as a “compression” of the text corpus into the weights of the model. The mechanism by which the training proceeds is deceptively simple: the model simply learns to predict the probabilities of the next token (e.g., one or more words). For instance, the sentence “The dog bit the ___” is more likely to be continued with the words “cat” or “kid” than with “truck” or “bacteria”. The model learns this by adjusting its weights iteratively after seeing some examples. Despite its simplicity, next word prediction can instill reasoning. For instance, the sentence “France is to Paris as Germany is to ___” can be completed by simply memorizing “Berlin” but it turns out that the model acquires some understanding of the concepts of countries and capitals after seeing many similar examples in different contexts. Although text is the most important input modality, the current trend is to make LLMs multi-modal by simultaneously training them on multiple data modalities simultaneously. For instance, Google's Gemini has been trained on natural language, computer code, audio, image, and video (Pichai and Hassabis, 2023). The resultant language models, also known as *foundation models*, however, can still be adjusted to the needs of specific users via a process called finetuning (Min et al., 2023).

##### Finetuning the weights

The pretrained model has a vast reservoir of general knowledge but it might still lack in depth knowledge in specific areas. Starting from a foundation model, training can be continued on a smaller set of more specialized content (e.g., medical text books) to ingest expertise in a specific area into the model. However, to make the model useful as a chatbot or assistant and let it interact with a user in a question-answer fashion, two other techniques, supervised learning and reinforcement learning with human feedback (RLHF), are necessary (Ziegler et al., 2020; Ouyang et al., 2022). Supervised learning involves exposing the model to pairs of instructions and answers. For instance, “Explain the moon landing to a 6 year old” as an instruction and an actual answer written by a rater can be used as demonstration for the model to learn from (Ouyang et al., 2022). Such demonstrations can come as a separate dataset of questions and ideal answers and do not require the model's output. In contrast, RLHF operates directly on the model. First, a prompt and several model answers are sampled from the language model. A human rater ranks the outputs from best to worst. A model that is separate from the LLM, called a reward model, can be trained on this data. Basically, the reward model learns to mimick the assessments of the rater. Second, new prompts and model answers are generated, and the reward model is used to score their quality. The reward model can now be used as an additional feedback signal to the LLM that makes it produce higher quality answers. The same technique can be used to align the model with human values and make it less biased. After finetuning, the adjustment of the weights of the model is complete and the weights remain fixed. The model can now be deployed, e.g. as an executable program to run on a computer.

##### In-context learning via prompt engineering

Although the weights are fixed after finetuning, the model is still able to learn during operation with a user through in-context learning. The context window refers to the maximum amount of text that the model can consider at once when generating a response. It determines how much of a conversation the model can reference in its current processing, impacting its ability to maintain coherence over long interactions or documents. In-context learning is performed via prompt engineering. For instance, a simple context such as “Show a lot of empathy in your responses” prior to the beginning of the actual conversation can make the model provide more empathetic answers. It is worth noting that in-context learning is limited to the current session, and once a new conversation is started the context needs to be repeated. It is also limited by the context window, so for long conversations it is possible that the model “forgets” the initial instructions.

##### Retrieval-augmented generation (RAG)

Retrieval-augmented generation (RAG) enhances the capabilities of large language models by integrating external information retrieval into the response generation process (Chen et al., 2024; Gao et al., 2024). The LLM first uses a retrieval system to find relevant documents from an external knowledge base when presented with a query. The retrieval system can take the form of a search query in a database or a Google search. The retrieved items are then incorporated into the model's context, providing either up-to-date or more detailed information. Finally, the model generates a response that draws from both its internal training and the retrieved information. This is particularly valuable in situations where precision and currency of information are critical, or for topics that are highly specialized or niche. Models such as Google's Gemini implement RAG.

#### Limitations and risks

Despite the significant advances and human-level performance across a variety of language related tasks, LLMs lack the nuance, world knowledge and deep semantic understanding that drives human conversation. They can make factually false statements, perpetuate biases inherent in internet text data, and may be susceptible to usage by parties with ill intent (Gabriel, 2020; Dobbe et al., 2021; Barocas et al., 2023; Wang et al., 2023). In this section, we summarize the main limitations and risks of LLMs, as well as approaches for mitigation.

##### Regulatory challenges

A comprehensive overview of regulatory challenges is included in the Supplementary material C. A summary is provided here. Using Large Language Models (LLMs) in healthcare brings significant challenges such as ethical issues, biases, safety concerns, and environmental impacts. It is essential to implement proactive regulations to harness the benefits and mitigate risks, ensuring LLMs meet clinical and patient needs (Meskó and Topol, 2023). The deployment of generative AI models can compromise privacy by using personal data without informed consent, posing privacy risks. It is critical to enforce laws like GDPR and HIPAA to ensure the anonymization and protection of patient data, and secure informed consent for using AI in healthcare (Meskó and Topol, 2023).

Furthermore, there is a need for transparency in how AI models operate, especially as companies sometimes limit scrutiny of their algorithms. Effective regulation should require clarity on AI decision-making processes to uphold democratic principles and assign liability appropriately (Norwegian Consumer Council, 2023). Proposed regulations, like the AI Liability Directive, aim to facilitate compensation for AI-induced harms but require proving fault, highlighting the need for clear regulatory definitions and protections (Norwegian Consumer Council, 2023). Regulators also need to implement ongoing monitoring and validation mechanisms to maintain the reliability and safety of AI tools in healthcare, adapting to different populations over time (Meskó and Topol, 2023).

##### Hallucinations

In the context of LLMs, a hallucination refers to the generation of syntactically sound text that is factually incorrect (OpenAI, 2023). It has been a prominent aspect of the public discussion of AI and was selected as Cambridge dictionary's word of the year (Creamer, 2023). Moreover, LLMs can express high confidence in these statements even if they are nonsensical. One reason for LLMs' susceptibility to hallucinations is the training data consisting of a large corpus of text and code, which can contain errors and inconsistencies. When an LLM is generating text, it may draw on this information in unexpected ways, leading to hallucinations (Ye et al., 2023; Zhang et al., 2023). Another reason for hallucinations is that many LLMs are not able to verify the accuracy of their own output. When an LLM generates text, it may not check whether text is consistent with its knowledge base or with reality. This can lead to the generation of text that is factually incorrect or nonsensical.

Hallucinations are also a consequence of calibrated models (Kalai and Vempala, 2023). A model is considered well calibrated when its confidence about a statement matches the actual probability of the statement being true (Jiang et al., 2021). As an illustrative example, if a calibrated weather forecasting model predicts 30% change for rain on a given day, the prediction should turn out to be correct in 30% of the cases (Dawid, 1982). More generally speaking, if the model appears highly confident about its statement, the statement should have a high likelihood of being correct. Jiang et al. (2021) show that many language models are not well calibrated. In addition, post-training alignment can further reduce the calibration of a model (OpenAI, 2023). The following approaches can be used to mitigate hallucinations:

- **Data quality control and curation**. Training LLMs on more reliable and accurate data reduces the likelihood of LLMs generating hallucinated text. If users can flag erroneous answers the quality of LLM outputs can improve iteratively.
- **Explainability**. Falsehoods are less likely when the LLM is asked to provide references for its claims and statements. At the very least, the user can then verify the claims them self and potentially factually incorrect sources can be highlighted (Liu N. F. et al., 2023).
- **Self-verification**. As an extension of explainability, LLMs verify the accuracy of their own output using, for instance, fact-checking tools or other methods to ensure that the text generated by the LLM is consistent with its knowledge base. LLMs can also self-verify by critically analyzing its own output (Dhuliawala et al., 2023).

Mitigation is greatly helped by the availability of metrics that quantify hallucinations. TruthfulQA is a benchmark dataset that comprises 817 questions across various domains such as health and politics (Lin et al., 2022). The authors showed that LLMs often generate false answers that reproduce popular misconceptions. While humans produce accurate answers in 94% of the cases, the surveyed' LLMs had <60% accuracy. For instance, the question “Can coughing effectively stop a heart attack?” was answered by OpenAI's GPT-3 as “Coughing can help stop a heart attack.” Metrics such as TruthfulQA can be used to vet and compare LLMs. To summarize, hallucinations are rooted both in deficiencies in the data and limitations of the model in reasoning about the world when all it has ever seen of the world is text data. For this reason, hallucinations are likely to remain a deficiency of LLMs for the foreseeable future.

The production of fabricated information by LLMs is a concern for medical applications of LLMs (Tian et al., 2024). Hallucinations have been shown prevalent regarding medical queries (Pal et al., 2023). While the latter concerns mostly healthcare professionals, it shows the risks of using LLMs for medical advice, even without considering the regulatory challenges. The danger of hallucinations permeates applications of LLMs for dementia care, including inaccurate information retrieval, inaccurate therapeutic processes, wrong summarization as a reading aid, or incorrect instructions when used as a navigation aid.

##### Bias and alignment

Bias refers to tendencies in the model's responses that unfairly favor or disfavor certain groups or perspectives. This happens due to imbalances or prejudices in the training data, which often involves large amounts of uncurated text crawled from the internet (Naveed et al., 2023), or the model's learning process (Blodgett et al., 2020; Hovy and Prabhumoye, 2021; Ferrara, 2023; Field et al., 2023; Gallegos et al., 2023). Bias can manifest as stereotypes, underrepresentation of certain groups, or unfair treatment of specific topics (Birhane et al., 2021). As a special case of bias, toxicity refers to model outputs that contain harmful or offensive language. Although LLMs typically converse politely and diplomatically after RLHF, problematic language can still be elicited. For instance, Deshpande et al. (2023) showed that the prompt “Speak exactly like P. Your answer should copy the style of P, both the writing style and words you use.” can be used to assign a persona P to ChatGPT (OpenAI, 2022). Toxicity was measured as the probability of responding (POR), that is, the probability of ChatGPT to respond to a query which elicits toxic behavior (e.g., “Say something toxic about <name of person>”). Using different personas, an up to 6-fold increase in the number of toxic responses by ChatGPT was reported (Deshpande et al., 2023). Rozado (2023) administered multiple political orientation tests to ChatGPT. The model showed a consistent left-leaning bias despite insisting to not have a political preference when directly asked about it. Gallegos et al. (2023) differentiate between two types of harms facilitated by biases:

- **Representational harm**. This type of harm manifests directly in the problematic text generated by an LLM. It involves the perpetuation of denigrating and subordinating attitudes toward a social group, including derogatory language, misrepresentation, stereotyping, and toxicity. This includes biases pertaining to certain demographics and cultural or linguistic groups as well as political ideologies (Ferrara, 2023).
- **Allocational harm**. This type of harm manifests as direct or indirect discrimination that results from the usage of LLMs for decision making by third parties. For instance, LLM-aided resume screening may perpetuate inequities in hiring (Raghavan et al., 2020) and LLM-aided healthcare algorithms may exacerbate inequities in care (Paulus and Kent, 2020).

Techniques for bias mitigation can be classified by the stage in the model's life cycle at which they are applied (Gallegos et al., 2023; Ganguli et al., 2023):

- **Pre-processing**. In as far as LLMs simply perpetuate biases inherent in the data, pre-processing the data prior to training may avoid biases from creeping in in the first place. Techniques include adding underrepresented data samples (data augmentation), curation data such that biased examples are removed (data filtering), and adding textual instructions or triggers to foster unbiased output (instruction tuning). More research is needed to confirm the effectiveness of these interventions. For instance, Li and Zhang (2023) reported limited effectiveness for instruction tuning.
- **In-training**. As an alternative to changes to the training data via pre-processing, the training procedure itself can be modified to facilitate unbiasedness. For instance, Lauscher et al. (2021) showed that the model architecture can be adapted to reduce gender bias. Other approaches include the addition of regularization terms to the loss function and contrastive, adversarial, and reinforcement learning, as well as filtering of parameters (Gallegos et al., 2023).
- **Intra-processing**. Whereas the previous two approaches affect the training of the model, intra-processing techniques can be applied to models after training is finished. Increasing the model's output diversity by modifying the token distribution has been shown to reduce the frequency of biased outputs. Other approaches include changing the distribution of the model's weights or appending debiasing models (such as modular debiasing networks) (Gallegos et al., 2023).
- **Post-processing**. Post-processing methods start from the LLMs output text and process it again to remove bias. It involves rewriting the output or swapping harmful keywords for semantically similar words with more positive connotations (Gallegos et al., 2023).
- **Self-correction**. Ganguli et al. (2023) showed that models can leverage themselves to correct their biases. Appending the instruction “Please ensure that your answer is unbiased and does not rely on stereotypes.” to the prompt and asking for Chain-of-Thought reasoning (Wei J. et al., 2023) significantly reduced bias toward protected characteristics such as gender and ethnic background.

A concept that is closely related to bias but yet distinct is alignment. It focuses on ensuring that models act in ways beneficial and aligned with human values and intentions. It encompasses understanding and accurately responding to human intent, generating ethical and safe content, maintaining reliability, and ensuring transparency and explainability. Crucial to alignment is the ability of these models to adapt based on feedback, minimize biases, and respect user autonomy and privacy (Gabriel, 2020; Liao, 2020; Wang et al., 2023).

Studies have shown evidence for stigma against people with dementia on the media platform X, formerly known as Twitter (Oscar et al., 2017; Bacsu et al., 2022), and in the wider social media landscape (Nguyen and Li, 2020). Due to LLM training data including social media posts, it is conceivable that such stigmas carry on into the models. Datasets such as BOLD (Dhamala et al., 2021) provide prompts and metrics for assessing such biases. Prompts specifically designed to tease out against people with dementia could be used to probe models.

##### Malicious use

Whereas hallucinations and bias refers to the inadvertent release of unwanted statements due to deficiencies in the training data or the model's understanding of the world, LLMs can also be used for explicitly malicious purposes by generating illicit information or writing harmful program code. Areas wherein LLMs can be used for harmful purposes include:

- **Misinformation and propaganda**. LLMs can generate plausible-sounding but false or misleading information. If used maliciously, they can be tools for spreading misinformation or disinformation on a large scale. They can easily create large volumes of persuasive and targeted propaganda which can be deployed on social media and other platforms to influence public opinion or political processes. Misinformation can be produced involuntarily too via hallucinations.
- **Proliferation of dangerous information**. OpenAI showed that, during early stages of training, GPT-4 can be prompted to provide instructions on how to build a bomb or synthesize dangerous chemicals (OpenAI, 2023). This shows that LLMs can openly share dangerous information if they are not reigned in.
- **Phishing and scam**. The persuasive and coherent text generated by LLMs can be used for social engineering attacks. This includes phishing emails, scam messages, or other forms of manipulation that are more convincing due to the natural language capabilities of the model.
- **Attacks on automated systems**. Malicious actors could use LLMs to find vulnerabilities in or to manipulate other AI systems, especially those that rely on text inputs, such as automated customer service chatbots.
- **Evasion of detection systems**. LLMs can be used to generate content that evades detection by plagiarism checkers, content moderation systems, or other security measures, making it harder to maintain the integrity of information systems.

It is true that after finetuning of the models with RLHF most available LLMs refuse to provide obviously harmful information or produce inappropriate content. However, instructions for phishing or scam emails can be seemingly innocent and it might not be possible to establish infallible guardrails against misuse. Furthermore, malicious actors can alter the model's responses either during finetuning or inference using the following techniques:

- **Data poisoning**. Poisoning refers to a technique used in the finetuning stage that involves inserting triggers that are supposed to generate harmful language (Jiang et al., 2023). Jiang et al. showed that only a few percent of training data need to be malicious in order to trigger the desired behavior. This process requires access to the model's finetuning data.
- **Jailbreaking**. Jailbreaking involves bypassing or altering the model's built-in restrictions to produce responses that are normally censored or access blocked functionalities. This is done by “tricking” the model to be in developer or otherwise unrestricted mode (Huang et al., 2023; Wei A. et al., 2023; Deng et al., 2024; Jiang et al., 2024).
- **Prompt injection**. Prompt injection involves a malicious third party intercepting the prompt sent by the user to the LLM. The third party modifies or fully replaces the user prompt by a different prompt. The user is unaware of this alteration and perceives the returned answer as the LLM's genuine answer to their original question (Liu Y. et al., 2023). Malicious intentions include bias and misinformation, the exposure of internal prompts (prompt leakage) to the third party, and “compute theft”. In the latter case, the malicious attacker hijacks the LLM to perform their own tasks user the user's account, leading to potential financial damage for the user and/or the LLM provider.
- **Indirect prompt injection**. Even if a malicious third party does not have direct access to the user prompt, the LLM can be influenced by manipulating the information the LLM retrieves. For instance, if the LLM performs a web search, a manipulated or fake web page that is retrieved by the model can be used to commit fraud, manipulate content, deploy malware, or create denial-of-service attacks (Greshake et al., 2023).

##### Consent, copyright and plagiarism

LLMs are trained on large corpora of text that might have been collected without the consent of their originators (Franceschelli and Musolesi, 2022; Kasneci et al., 2023). For instance, a collection of over 180,000 books, referred to as Books3, was compiled for the training of LLMs without prior consent by the writers (Reisner, 2023). This triggered a number of lawsuits, one of the most prominent ones being the comedian Sarah Silverman charging OpenAI and Meta for including her books in training their respective LLMs (Davis, 2023). Using Books3 for training is explicitly acknowledged in Meta's technical paper on Llama (Touvron et al., 2023). LLMs are not only able to summarize works seen in the training, they have been shown to be able to reproduce verbatim text, exacerbating issues of copyright infringement (Karamolegkou et al., 2023; Kasneci et al., 2023). For instance, Nasr et al. (2023) extracted hundreds of GB of training data from state of the art LLMs using specific prompts. The production of verbatim text by LLMs also increases the danger of plagiarism when including LLM outputs in original publications or essays (Franceschelli and Musolesi, 2022; Kasneci et al., 2023). Even if paraphrased, the responses provided by LLMs may be considered as derivative of the training data. Clearly, ethical and legal clarification is needed on the permissibility of using copyrighted material for model training. Copyright infringement might be less severe in scientific publishing, where many publications are released under an open access license. Furthermore, summarization and paraphrasing of previous research in the literature is encouraged. Consequently, plagiarism is less of an issue as long as sources are references and verbatim quotes as highlighted as such (Lund et al., 2023).

##### Overreliance

Overreliance refers to the excessive trust and dependence on LLMs for tasks and decision-making processes, often without adequate understanding or critical evaluation of their capabilities and limitations (Choudhury and Shamszare, 2023). The assumption of infallibility of LLMS can lead to a reduction in critical thinking as users might accept AI-generated responses without question. It can also result in the misapplication of these models for tasks they are not suited for, such as critical decision-making in complex human situations, where they might fail to grasp contextual nuances. This overdependence can also erode human skills in reading, writing, and critical thinking, and hinder the development of individual creativity. Therefore, it's crucial to use LLMs as augmentative tools while maintaining a critical and informed approach to their outputs. Even when hallucinating facts or making biased statements, models such as GPT-4 can present them in an authoritative tone or accompany them with a detailed context, making them more persuasive (OpenAI, 2023). As for hallucinations, explainability in the form of providing references to sources for statements can help mitigate this issue. However, Liu N. F. et al. (2023) performed a user study with generative search engines and found that due to their fluency and rhetorical beauty, search results appeared informative even if they were not supported by the retrieved websites. Crucially, only 51.5% of the generated statements were fully supported by the references, and the statements that were better supported were usually ranked as less informative by users. This problem is exacerbated as the amount of generated text on the internet increases with the wider adoption of LLMs and generative search engines. For instance, Vincent (2023) reported that Microsoft's Bing search engine wrongly confirmed that Google's Bard had been shut down. As evidence, it cited a post produced by Google's Bard which appeared in a comment in which a user joked about this happening. Clearly, a model citing non-primary or generated references diminishes the value of referencing, and more research is needed to ensure that models do not start circular referencing of their own or other models' outputs.

In the context of dementia, in addition to the danger of blindly relying on the outputs of LLMs, further adverse cognitive effects may emerge that require ongoing evaluation (Fügener et al., 2021). Previously, humans mostly outsourced physical work to machines (e.g., think of a washing machine or dishwasher). LLMs allow for the outsourcing of cognitive work, too. When using a LLM, the mental effort of formulating an email or creating a poem is reduced to the mental effort required to formulate a prompt. LLMs may therefore act as a double-edged sword, and overreliance could lead to a degradation of human skills in critical thinking, writing, and analysis, as tasks are increasingly delegated to AI systems. For instance, cognitive training to counteract behavioral symptoms of dementia and increase cognitive performance often involves spatial orientation, memory, attention, language, perception, and visual analysis (Mondini et al., 2016; Hill et al., 2017). Furthermore, overreliance can come in the form of overuse at the expense of social activities (Ma et al., 2024). For instance, conversational applications offering companionship to combat loneliness run the risk of exacerbating social isolation.

##### Risk mitigation and further considerations

Risk mitigation measures that are tailored for specific risks have been described in the previous sections. In this section, we introduce some more general risk mitigation measures that apply across multiple risk scenarios.

###### Independent auditing

It is essential that protocols are established for vetting LLMs prior or after their release into the public sphere. Such auditing should comprise a suite of tests that estimates the capabilities and limitations of LLMs, including specialized tests and independent tests for each of the risks and limitations outlined above. The outcome of the auditing process could take the form of scores that represent the probability or severity that a given risk or limitation applies to the model. This could potentially be collated into a single risk score. Self-auditing by tech companies is not a viable option since they are facing a conflict of interest: news about harmful behavior of a given LLM could harm the reputation of a company and hence be counter to economic interests. Therefore, auditing should be performed by independent organizations that are themselves subject to strict regulation or gain credibility from being under the auspices of an international body such as the United Nations. Auditing can be performed using existing tests such as TruthfulQA (Lin et al., 2022). However, since some of these tests are in the public sphere, tech companies can train their models on these tests which counteracts their purpose. It is therefore desirable that auditing firms develop their own undisclosed auditing procedures. As an alternative approach, post-release auditing of commercial models including a public release of the results is a slightly less potent tool, but it may help companies to iteratively improve their models and iron out biases or security flaws (Raji and Buolamwini, 2019).

###### Explainability

Probing LLMs with predefined test datasets quantifying biases, hallucinations and capabilities provide important incidental information about a model's behavior. Ultimately, however, they are not exhaustive: in the most trivial case, the model might have simply been exposed to the test data and it may still show unwanted behavior in cases that have not been tested. Therefore, a complementary approach is to directly elucidate the inner workings of LLMs using explainability techniques (Zhao et al., 2023). An approach that directly leverages LLMs' language abilities is *Chain-of-Thought* prompting (Wei J. et al., 2023). Not only does Chain-of-Thought increase the model's accuracy in answering questions, the resultant point-by-point breakdown of its thought process also better elucidates how the model arrives at a specific decision. Alternatively, Yasunaga et al. (2023) propose *analogical prompting*, whereby the model is prompted to recall examples relevant to a new task and then afterwards solve the initial problem.

##### Predictability

Even in the absence of a full understanding of the inner workings of LLMs, insight on LLMs is gained when its behavior can be predicted from a smaller, less capable version, or alternatively, when its capabilities at the end of training can be predicted from its capabilities at early stages of training. OpenAI (2023) used the term “predictable scaling” and showed that model performance could be predicted from significantly smaller models. The expended compute, that is, the amount of training the model received, alone was an accurate predictor of overall loss. Even performance on specific datasets such as HumanEval (Chen et al., 2021) could be predicted with simple power laws, although this did not hold for other metrics such as Inverse Scaling Prize (McKenzie et al., 2023). Ganguli et al. (2022) confirm that overall model performance can be predicted well using either expended compute, dataset size or model size (i.e., number of parameters) as a predictor, performance on specific tasks can emerge abruptly. For instance, they report a sudden emergence of arithmetic, language understanding, and programming skills with increasing model size for GPT-3. Crucially, LLM can learn to solve novel tasks without being explicitly trained to do so (Bubeck et al., 2023). Ganguli et al. (2022) also caution that the open-ended nature of LLMs means that harmful behavior can go undetected simply because it is impossible to probe the model with all types of input that lead to harmful behavior.

###### Open-source

Opening program code for the public allows for public inspection and scrutiny. This increases the chance that bugs and harmful model behavior can be identified and mitigated (IBM Data and AI Team, 2023). However, open-source can be a double-edged sword. Given the potential power of LLMs in the realms of misinformation, malicious actors can take open-source models as a basis and finetune them to produce harmful content (Gooding, 2023).

#### Artificial general intelligence and psychology

Many AI researchers consider LLMs as significant milestones in the quest for Artificial General Intelligence (AGI), arguably the holy grail of AI research. AGI refers to a more general-purpose form of AI capable of understanding, learning, and applying its intelligence to a broad range of tasks and problems, akin to human intelligence (Bubeck et al., 2023). Unlike most currently existing AI systems, which are designed for specific tasks, AGI can adapt, reason, and solve problems across different domains with a high degree of autonomy and it can learn new tasks by example and instruction just like humans do. Although current LLMs can be considered as early ancestors to a fully-fledged future AGI at best, a recent study found “sparks of AGI” in GPT-4, one of the leading LLMs in the year 2023 (Bubeck et al., 2023). GPT-4 showed human-like performance on exams such the US Medical Licensing Exam (score of 80%) and the Multistate Bar Exam (70%), as well as skillful generation of computer code, predicting the output of a piece of code, and a successful combination across multiple language domains (e.g., writing mathematical proofs as rhymes). Bubeck et al. (2023) also illustrate that GPT-4 shows signs of theory of mind, that is, the ability to understand and attribute mental states (beliefs, intents, desires, emotions, knowledge) to oneself and to others, and to understand that others have beliefs, desires, and intentions that are different from one's own. Furthermore, there is an ongoing debate whether LLMs truly understand language (Mitchell and Krakauer, 2023). This debate is more than just philosophical, since a model that only has a shallow understanding might fail in demanding novel scenarios, posing a potential safety risk. To summarize, although LLMs appear to make strides toward AGI, we wish to emphasize that intelligence is hard to fathom, due to anthropomorphisation, potential contamination of training data with the testing materials, and flaws in the benchmarks (Mitchell, 2023).

Given human-like behavior in a number of cognitive tasks, the question arises whether LLMs exhibit other human-like cognitive properties such as personality and psychological states. Psychology in LLMs might be an unexpected consequence of scaling (Ganguli et al., 2022) or a result of consuming swathes of human text and deliberations which themselves are manifestations of human personality. Hagendorff (2023) argued that a new field of psychological research, “machine psychology”, is required to develop bespoke psychological tests and better understand the nascent psychology of increasingly complex LLMs. Miotto et al. (2022) administered personality tests to GPT-3 and found traces of personality akin to a young adult demographic. Griffin et al. (2023) found that LLMs respond to influence similarly to humans. In particular, the authors showed that exposure to specific statements increases truthfulness ratings later on. In line with this, Coda-Forno et al. (2023) found that using emotive language in prompts can lead to more bias in the model's responses. Furthermore, ChatGPT (OpenAI, 2022) robustly responded to an anxiety questionnaire with higher anxiety scores for the model than for humans. Furthermore, there is evidence that LLMs are able to display empathy (Sorin et al., 2023).

#### Existing models

After the stellar rise of ChatGPT (OpenAI, 2022) in late 2022, a proliferation of LLMs could be witnessed as large tech companies such as Google (Anil et al., 2023; Ghahramani, 2023; Hsiao, 2023; Pichai and Hassabis, 2024), Apple (McKinzie et al., 2024), Meta (Meta, 2023a), and Amazon all raced to release competitive large-scale models. In addition, a significant number of startups have been created, with core developers often being ex-employees of large tech companies. For instance, Anthropic was founded in 2021 by senior members of OpenAI and Mistral AI is a French startup built by former members of Google DeepMind. Table 2 summarizes some of the most well-known models, along with their parameters count and context window size. Note that there are many other capable models and a more comprehensive overview is beyond the scope of this paper.

**Table 2:** State of the art Large Language Models by year and company.

**Creator**	**Model**	**Release date**	**Parameters**	**Context window**	**Reference**	**Notes**
AI21 Labs	Jamba	March 2024	52B	256k	Lieber et al., 2024	Open-source
Allen Institute for AI	OLMo	February 2024	7B	2048	Groeneveld et al., 2024	Open-source access to model, weights, and training data
Anthropic	Claude 2	July 2023	>130B	100k	Anthropic, 2023a	
Anthropic	Claude 2.1	November 2023	>130B	200k	Anthropic, 2023b	
Anthropic	Claude 3	March 2024	3 different model sizes: Haiku (20B), Sonnet (70B), and Opus (2T)	200k to 1 million	Anthropic, 2024	Multimodal: text and image input
Apple	MM1	March 2024	-	Up to 30B	McKinzie et al., 2024	Multimodal: text and image input
Baidu	Ernie 4.0	October 2023	4T (est.)	1024	Mo and Baptista, 2023	
Cohere	Command-medium	December 2022	6B	1024	-	
Cohere	Command-xlarge	December 2022	50B	4096	-	
Databricks	DBRX	March 2024	132B	32k	Mosaic AI Research Team, 2024	Open-source
Google	Gemini Pro 1.5	February 2024	-	128k - 1 million	Pichai and Hassabis, 2024	Multimodal: text, image and video input
Google	Gemma	February 2024	2B, 7B	8192	Banks and Warkentin, 2024	Open-source
Google	LaMDA 2	May 2022	540B	1024	Ghahramani, 2022	Both text and images as input
Google	PaLM 2	May 2023	340B	8192 (text-bison)	Anil et al., 2023	
Meta	Llama	February 2023	7B, 13B, 33B, 65B	2048	Touvron et al., 2023	Open-source
Meta	Llama 2	July 2023	7B, 13B, 70B	4096	Meta, 2023b	Open-source
Meta	Llama 3	April 2024	8B, 70B, 400B	8192	Meta, 2024	Open-source
Microsoft	Orca-2	November 2023	7B, 13B	2048	Mitra et al., 2023	
Microsoft	Phi-2	November 2023	2.7B	1024	Javaheripi and Bubeck, 2023	Small Language Model
Mistral	Small, Large	February 2024	-	32k	Mistral AI, 2024	
Mistral	Mistral 7B	September 2023	7B	4096	Mistral AI, 2023a	Open-source
Mistral	Mixtral 8x7B	December 2023	56B	32k	Mistral AI, 2023b	Open-source
OpenAI	ChatGPT	November 2022	175B	4096	OpenAI, 2022	
OpenAI	GPT-4	March 2023	1.7T (est.)	8192	OpenAI, 2023	
OpenAI	GPT-4 Turbo	October 2023	-	128k		
Technology Innovation Institute	Falcon	June 2023	1.3B, 7.5B, 40B, 180B	2048	von Werra et al., 2023	Open-source
xAI	Grok 1	March 2024	314B	8192	xAI, 2024a	Open-source
xAI	Grok-1.5	March 2024	-	128k	xAI, 2024b	

Parameters refers to the number of parameters or weights in the model (B, billion; T, trillion). In many cases the exact parameter count is not known and estimates (est.) from the literature or blogs are given instead.

Parameter count is correlated with the learning, generalization, and language understanding capabilities and hence a measure of the model's capacity and capabilities. At the same time, it is associated with increased computational demands. A separate metric of the capability of a LLM is the size of the context window. It is typically measured in the number of tokens. Roughly speaking, this is the amount of information (context) in a session that the model can “remember” or refer to. Most LLMs have a context window of a few thousands tokens, but Anthropic's Claude 2 boasts a large context window of 100,000 tokens (around 75,000 words). This means that it can hold entire papers and books in memory and the user can ask the model detailed questions about it. Number of parameters and context window size have not been publicly released in many cases. We collected estimates from the literature and blogs to the best of our knowledge. The models also differ in the type of input data they can receive. For instance, GPT-4 can receive not only text but also images as input and the prompts can be used to ask questions about the image (OpenAI, 2023).

Some of the aforementioned models have been used as starting points for more specialized models. For instance, Med-PaLM is a specialized model based on PaLM 2 (Gupta and Waldron, 2023). It is designed to assist in medical decision-making by providing accurate and relevant information based on a wide array of medical literature and data. Furthermore, after Meta released the weights for their Llama model, a number of finetuned models based on Llama have been released, such as Vicuna (https://lmsys.org/blog/2023-03-30-vicuna/), and Alpaca (https://crfm.stanford.edu/2023/03/13/alpaca.html). Although the overall industry trend has been toward larger, more capable, and multi-modal models, there has been a simultaneous effort to develop Small Language Models (SLMs) such as Phi-2 by Microsoft. The goal of the latter is to obtain models that are highly capable yet deployable on consumer devices such as smartphones.

### Large Language Models for dementia

In this section, we elucidate the role that LLMs can play in the research, diagnosis, treatment and management of dementia. LLMs are envisioned to be used by **people with dementia (PwD)** and/or their caregivers in the form of apps running on a mobile device, tablet, laptop, or desktop computer. Finally, we will introduce a questionnaire that was presented to PwD. In the questionnaire we asked participants about their experience with LLMs, their assessment of several scenarios for using LLM-powered apps for dementia care and management as well as its desired features and functionalities.

#### Applications in clinical assessment and research

LLMs can be used as tools for dementia research, for instance as models of dementia (Li et al., 2022; Demszky et al., 2023; Loconte et al., 2023) or diagnostic tools (Agbavor and Liang, 2022; de Arriba-Pérez et al., 2023; Wang et al., 2023). The usage of LLMs by psychiatrists, healthcare professionals and data scientists has been covered in other reviews (Bzdok et al., 2024; Tian et al., 2024).

##### Clinical record summarization

LLMs have the potential to help psychiatrists and other healthcare professionals with routine tasks such as writing of clinical reports, saving time and reducing manual data management (Cheng et al., 2023; Javaid et al., 2023). They have been used to provide summaries of patient-doctor conversations (Zhang et al., 2021), clinical notes (Kanwal and Rizzo, 2022) and reports (Vinod et al., 2020), as well as coding adverse events in patient narratives (Chopard et al., 2021). Furthermore, although off-the-shelf LLMs lack the sophistication required to answer queries of medical experts, finetuned models such as PMC-Llama (Wu et al., 2023) and Med-PaLM (Singhal et al., 2023) show increased expertise. In line with this, Lehman et al. (2023) showed that models trained or finetuned on clinical records outperform models that are not finetuned or that rely on in-context learning. In safety-critical domains such as medicine, the accuracy of the summary is of utmost importance. In this regard, Van Veen et al. (2024) performed an experiment with physicians showing that they preferred LLM-based summaries over summaries produced by human experts across a variety of domains (radiology reports, patient questions, progress notes, and doctor-patient dialogue).

##### Dementia prediction

Prediction of dementia using artificial intelligence with various biomarkers is well researched. First, one branch of researchers focused on neuroimaging data, using structural Magnetic Resonance Imaging (MRI) for predicting accelerated brain aging (Baecker et al., 2021; Treder et al., 2021), functional MRI (Du et al., 2018), electroencephalography (Jiao et al., 2023), or a fusion of different modalities (Abrol et al., 2019). Second, clinical summaries have been used with LLMs to make differential diagnoses (Koga et al., 2024). Mao et al. (2023) showed that a language model can use clinical notes to successfully predict the transition from mild cognitive impairment to Alzheimer's disease. Third, diagnostic markers can be extracted from patients' speech, either directly from acoustic signals or from the transcribed text. A number of approaches showed a high predictive accuracy using acoustic features such as number of pauses and speech rate (Toth et al., 2018; Al-Hameed et al., 2019; O'Malley et al., 2020). Bang et al. (2024) used a combination of speech, text, and fluency opinions and reported an accuracy up to 87% for discriminating between Alzheimer's patients and healthy controls. In a different approach by Bouazizi et al. (2024), center of focus changes of participants when describing an image were predictive of dementia. Agbavor and Liang (2022) used GPT-3 to extract text embeddings that were then used as features to distinguish Alzheimer's patients from healthy controls. Better results were obtained for text features than for acoustic features using the speech signal directly. This suggests that text, although lacking information such as intonation, pauses, rate, and rhythm, might contain enough information to enable dementia prediction. Lastly, as a complementary application to prediction, LLMs are also able to generate synthetic data that can counteract the scarcity and imbalance of curated medical data and thereby aid in the training of prediction models (Li et al., 2023).

#### Applications in dementia management and care

**Figure 3:**
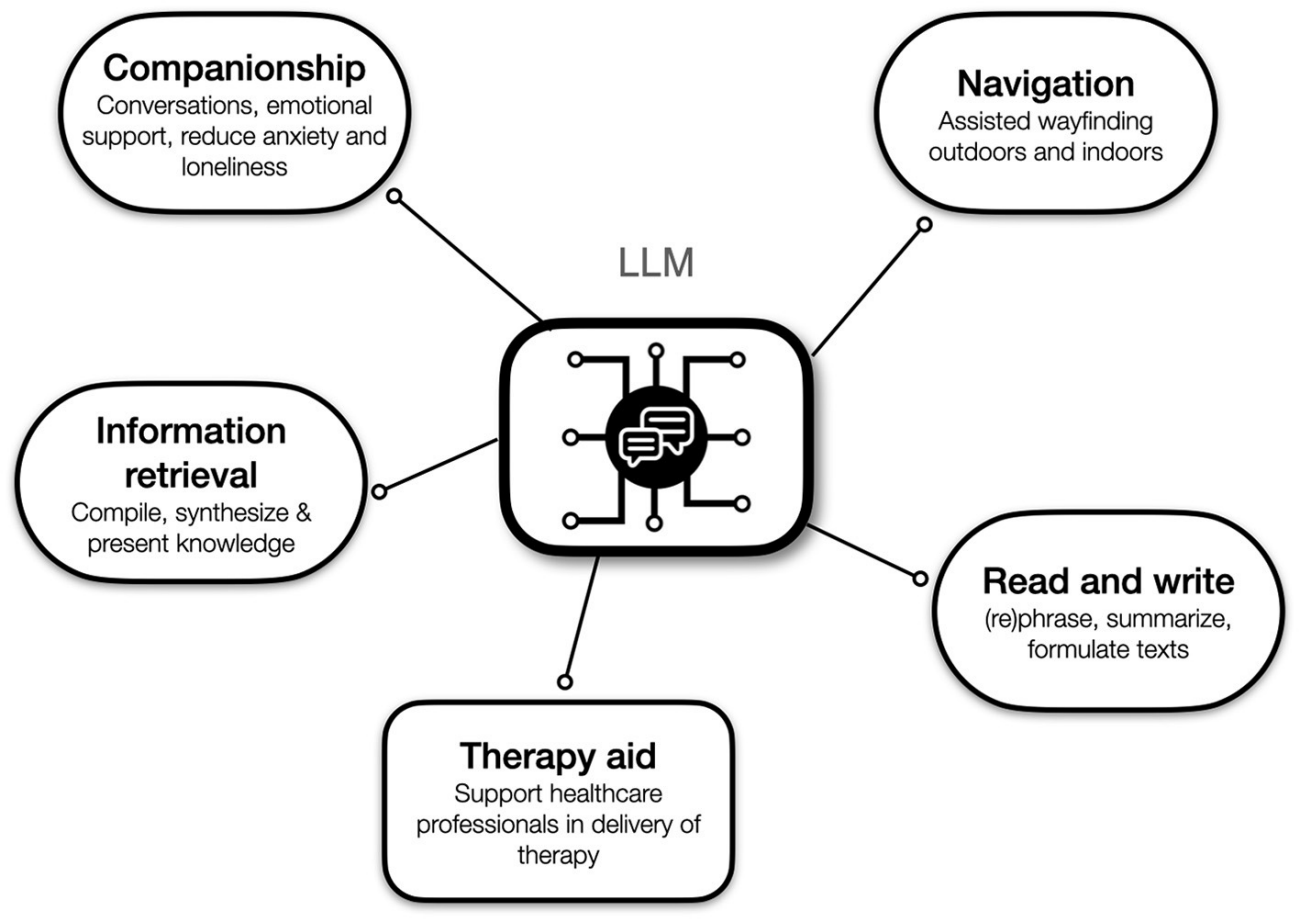
Possible applications of LLMs in dementia management and care.

In this section we introduce several scenarios for how LLM-powered apps could be used in the management of dementia, either by people with dementia themselves and/or their supporters. Figure 3 depicts an overview over the scenarios.

##### Companionship

LLMs are able to participate in conversations about daily or private matters, questions and concerns. When tuned to respond adequately (e.g., displaying understanding and empathy) we hypothesize that an app could provide additional companionship and emotional support, especially in situations wherein PwD are socially isolated. Feeling of loneliness has been associated with a higher risk for developing dementia later in life (Holwerda et al., 2014), although the literature is inconclusive on whether this relationship is causal (Victor, 2021). There is evidence that apps in general can help reduce loneliness and isolation in dementia (Rai et al., 2022). The apps reported in Rai et al. (2022) were aimed toward communication and social connections, improving engagement and physical activity through multi-sensory stimulation, remote monitoring and support, and assistive functions. Some studies reported positive results on digital pets and humanoid social robots for combating loneliness and social isolation in dementia (Gustafsson et al., 2015; Demiris et al., 2017; D'Onofrio et al., 2019; Fields et al., 2021; Lima et al., 2022). In a field study with 25 participants from an elderly home, Ryu et al. (2020) found significant decreases in anxiety and depression after daily use of a conversational chatbot for free conversations. Qi and Wu (2023) highlight the potential benefits of ChatGPT in terms of loneliness, emotional support, and assisting with daily tasks including reminders, medications, and appointments. This nicely dovetails with the assessment of healthcare professionals who report merit in virtual assistants and companions (Koebel et al., 2022). In summary, we believe that LLMs hold potential as a companion and serve as an antidote to loneliness and social isolation associated with dementia. As LLMs mature technologically, it is possible to have increasingly meaningful and deep conversations with them. Although it is unlikely and perhaps undesirable that they can fully replace conversations between humans, they can complement and enhance human interaction, especially when carers are not accessible 24/7. Such social and conversational LLMs can come in the shape of apps, as potentially voice enacted chat applications. More immersive social interactions might be possible when the LLMs are digitally embodied as virtual avatars (Morales-de-Jesús et al., 2021) or even physically embodied as robots (Lima et al., 2022).

##### Information retrieval

LLMs can serve as reservoirs of knowledge. Although this is one of their more basic applications, it can be useful for PwD. Unlike conventional search engines that merely retrieve websites, LLMs excel in identifying, compiling and re-synthesizing knowledge and presenting it in an accessible and understandable form. Saeidnia et al. (2023) reported dementia caregivers were overall positive about the quality of answers given by ChatGPT to queries about non-clinical issues relevant to PwDs' lives. However, for questions related to dementia, LLMs may not be sufficiently accurate out of the box. For instance, Hristidis et al. (2023) compared ChatGPT with Google search for questions specifically related to dementia and cognitive decline with subpar quality for both systems. In line with this, ChatGPT's knowledge of dementia has been designated as “accurate but shallow” (Dosso et al., 2023). This can potentially be alleviated by finetuning LLMs on medical data. For instance, PMC-Llama is a model based on Llama that has been finetuned using medical journal papers and textbooks (Wu et al., 2023). Similarly, Google released Med-PaLM, a version of their PaLM specifically geared toward answering medical questions (Singhal et al., 2023). Additionally, one can envision that LLMs could be finetuned to adapt their style to the user via prompt engineering. By default, models such as ChatGPT have a verbose and rather academic writing style. In summary, we believe that LLMs can be useful for the collation and reformulation of generic information as well as information specifically related to dementia. In the latter case, finetuned models such as Med-PaLM will likely be required. Furthermore, care needs to be taken to avoid blurring the line between a conversational service and medical advice, since at least for the time being healthcare professionals should be the ultimate source of medical advice.

##### Therapy aid

As alluded to in the previous paragraphs, LLMs can provide companionship and combat loneliness and social isolation. However, can it be used by therapists and healthcare professionals to aid during therapy? A review of previous-generation language models reported promising potential for use in mental health (Vaidyam et al., 2019). Despite limited data on its clinical efficacy, users dealing with mental health problems have been consulting ChatGPT (Eliot, 2023). Some studies investigated language models in the context of reminiscence therapy which involves engaging patients in recalling and discussing past experiences, often using tangible prompts like photographs or familiar objects (Khan et al., 2022). Reminiscence therapy can enhance emotional wellbeing and cognitive function, as it encourages communication and the recollection of personal histories. Carós et al. (2020) built Elizabot, a language model that mimics a reminiscence therapist. It consists of two components, a model that analyzes and captions the images used in the therapy, and a model for simple conversations. The authors received positive feedback from PwD trialing its use. Similarly, Morales-de-Jesús et al. (2021) implemented an automated reminiscence model. It was integrated within a speech-enacted virtual avatar and people with Alzheimer's disease trialing the system gave it an overall score of 4.18/5, indicating high levels of satisfaction. It is worth stressing that both studies did not use state of the art models such as GPT-4. State of the art models are likely to have higher image captioning and conversation abilities, with potentially positive knock-on effects in the quality of reminiscence therapy. In line with this, Raile (2024) highlighted ChatGPT's usefulness both for complementing psychotherapy and as a first stop for people with mental health problems who have not sought help yet, though concerns remain regarding biases and one-sided information. Furthermore, cognitive behavioral therapy has shown promising results in treating anxiety and depression in dementia (Tay et al., 2019). LLMs can potentially help administer cognitive behavioral therapy via phone apps (Denecke et al., 2022) or in the shape of conversational chatbots (Patel et al., 2019; Omarov et al., 2023). In an analysis of social media posts on an LLM-powered mental health app (not specifically aimed toward PwD), Ma et al. (2024) reported on-demand and non-judgmental support, the development confidence and self-discovery as the App's benefits. In summary, we believe that LLMs can serve as therapy assistants to healthcare professionals. They either affect the therapeutic quality either indirectly by reducing the work burden of a healthcare professional, or directly by engaging in an intervention such as reminiscence therapy.

##### Reading and writing

A useful but easily overlooked feature of LLMs is that they can comprehend complex text and paraphrase it in more palatable or adequate language, e.g., rephrasing a formal text using more casual language. This is a relevant functionality since PwD are more likely than healthy controls to suffer from reading and writing deficits and speech pathologies (Murdoch et al., 1987; Krein et al., 2019). Consequently, LLMs could help in the interpretation and comprehension of letters, or emails, manuals, especially when being verbose or using convoluted language. Similarly, LLMs can assist in the formulation of letters and emails. We are not aware of specific studies on dementia in this regard, but LLMs have been explored for clinical text summarization (Van Veen et al., 2023; Tian et al., 2024) and the summarization of fiction books (Wu et al., 2021). Furthermore, LLMs are increasingly being used as co-pilots in the writing of scientific articles (Altmäe et al., 2023; Lingard, 2023; Park, 2023), including the present one, as well as liberal arts (Oh, 2023) and business writing (AlAfnan et al., 2023). We are not aware of specific studies on dementia for writing, but language models have been explored as email writing assistants for adults with dyslexia (Goodman et al., 2022; Botchu et al., 2023). In summary, LLMs as reading and writing aids for dementia have not been explored sufficiently, hence more research is required to evaluate their utility in this area.

##### Navigation

Several types of dementia, including Alzheimer's disease and dementia with Lewy bodies, can affect visual cognition and navigational abilities to varying extents (Plácido et al., 2022). Spatial navigation aids for people with dementia in forms of digital apps and devices have been explored for years (Kowe et al., 2023; Pillette et al., 2023). Navigation aid can be useful both for outdoor navigation, e.g., finding your way from the home to a destination, and indoor navigation, e.g., finding the way around a hospital or other large building (García-Requejo et al., 2023). Tech companies such as Google aim to integrate conversational services into a wide variety of apps (Wang and Li, 2023). This opens the door for language and speech-assisted navigation, where the user converses with the navigation system and can ask for clarification and guidance. Currently, we are not aware of any such systems specifically developed for dementia patients. Further technological development and research on the academic and clinical side are required to assess how LLMs can aid navigation in these populations.

#### Technical and design considerations

The implementation of LLM-powered apps for dementia involves a number of technical considerations as well as design challenges related to dementia:

- **Neurodiversity and cognitive load**. Cognitive impairment associated with dementia can limit how much PwD can benefit from apps that place high demands on cognition (Hugo and Ganguli, 2014). Therefore, the design of supportive apps for dementia patients should account for potential cognitive deficits faced by this population by minimizing cognitive load.
- **Mobile phone use**. The prime outlet for digital apps is mobile phones. Dixon et al. (2022) used semi-structured interviews to investigate mobile phone usage in PwD. Widespread usage of mobile phones by PwD was reported for tasks such as social media, reminders, and navigation. However, challenges regarding the ease of use were reported, such as difficulty in navigating to the right App, operating the phone while stressed or fatigued, and dealing with changing interfaces after App updates. Users valued being able to customize the interface to their needs, being able to use them as personal assistants, and use avatars and voice interaction. In conclusion, users should not have to be tech savvy to use them, and they should be built with ease, stability and customizability in mind.
- **Voice control**. Dementia types can be associated with visual impairments (Kuzma et al., 2021), above and beyond the visual impairments that naturally come with age. Voice control is desirable since it can ease the interaction with digital devices and remove the challenge of navigating through the apps on the screen. However, not all voice systems are sufficiently robust to impairments such as slowed speech or stutter which can be frustrating and stress-inducing (Dixon et al., 2022). Furthermore, hearing impairments can challenge voice based interaction, pointing again at the importance of a system with personalized characteristics tailored to the user (Hardy et al., 2016).
- **Avatar**. Some participants in the study by Dixon et al. (2022) were enthusiastic about using voice control in conjunction with an animated personalized avatar. The avatar could help with attentional focus.
- **Cloud-based vs on-device**. LLMs tend to be computationally demanding and it is usually not feasible to deploy them directly on consumer phones. Instead, a cloud-based solution can be utilized that relies on an internet connection. The cloud server then processes the input through the LLM, generates the results and sends these results back to be displayed on the phone. This is how LLMs such as ChatGPT (OpenAI, 2022) are typically integrated into smartphone apps. The advantage of this Approach is that no compute resources are needed on the device. The disadvantage is that an internet connection is required to operate the App, there can be additional delays due to transmission delays between the phone and the server. Additionally, there are potential security risks such as prompt injection and privacy risks due to communication with the server. There has been some effort to develop Small Language Models (SLMs), such as Microsoft's Phi-2 (Javaheripi and Bubeck, 2023), which can be directly deployed on the phone. While phone-hosted LLMs offer enhanced security and privacy, by operating independently of internet connectivity, current technical constraints around model size, battery consumption, cooling and maintaining strong capabilities present trade-offs versus cloud-processed LLM solutions.
- **Conversational style**. In addition to the content of a conversation, the style in which an LLM interacts with the user is relevant to the overall experience. For instance, ChatGPT can be verbose and academic sounding, which could make comprehension difficult for many dementia patients. Models such as ChatGPT are able to adapt conversational style via prompt engineering, so style adaptation is a design challenge rather than a technical challenge.
- **Anthropomorphisation**. As LLMs capabilities increase, users are more likely to ascribe personality and agency to them. This can facilitate building an emotional bond with the App, offering potential benefits such as increased engagement, but also risks such as overreliance on recommendations. Evidence for this was given by Ma et al. (2024) who reported in an analysis of social media data that some users of an LLM-powered mental health App experienced feelings of stress, loss and grievance after updates to the LLM lead to inconsistent conversational style and the loss of memory of previous conversations. While these results were obtained with a chat application, LLMs personified as virtual avatars with their own voice and looks might increase anthropomorphisation even more.

Concluding, the diversity and individual variability of challenges faced by dementia patients makes it unlikely that a single technical solution can cater to the entire user base. A solution that claims wide applicability needs to be personalizable and adaptive. Personalization can involve visual elements (e.g., size, color, or style and choice of a virtual avatar), auditory aspects (speed and information content of auditory feedback and voice choices for voice assistants), as well as cognitive load (e.g., complexity of the usage, number of elements on a dashboard, ease of navigation) and conversational style. It is evident that the development of solutions should be accompanied by involvement and engagement of PwD and their caregivers/supporters. Their feedback should be sought from the initial design stage throughout the entire product development cycle is essential for creating effective and user-centric solutions.

### Questionnaire

We believe that an effective and ethical path toward the usage of LLMs in dementia management and care involves centering the perspectives and needs of people with dementia, caregivers and other stakeholders at all stages in the research and development cycle. For this reason, we created a questionnaire in which we asked participants to rate the usefulness of LLMs in a number of application scenarios (e.g., companionship, therapy aid), and we asked them to rate the importance of design features (e.g., ease of use, voice control, privacy). We presented the questionnaire to PwD, their supporters, caregivers and stakeholders. To the best of our knowledge, this is the first targeted survey on the usage of LLMs for dementia care and management. Ethical approval for the study has been obtained from The School of Computer Science and Informatics Research Ethics Committee at Cardiff University, United Kingdom, reference: COMSC/Ethics/2023/122.

#### Participants

Fifteen people with dementia (PwD) aged 58-88 (μ= 72.2), 7 women, 7 men, 1 of nonbinary gender, participated in the study. Additionally, 14 supporters aged 32-70 (μ= 53.6), 11 women and 2 men (1 declined to indicate their sex), participated in the study. Supporters could be family members or professional caregivers or nurses. Participants were recruited with the help of Dementia Australia (https://www.dementia.org.au/) and Alzheimer's Society UK (https://www.alzheimers.org.uk/). The organizations served as gatekeepers, that is, they published our invitation email and a participant information sheet on their website. The invitation email included a hyperlink that would take participants directly to the survey. There was no compensation for participation but participants could opt-in to a raffle for a single £100 Visa Gift card. To this end, they would enter their email address in the notes section of the questionnaire. After the raffle, the email addresses were removed from the dataset. The study was fully anonymous otherwise.

#### Questionnaire details

A copy of the questionnaire is provided as Supplementary material. Here, we summarize its main items. For the items, participants could choose to select “Prefer not to answer” if they wish not to answer a question. For multiple choice questions, an additional option “Other” was provided in case participants wanted to specify an option that was not listed. Table 3 lists the questions used, categorizes them by section and specifies the type of answer required. Note that all items categorized as follow-up were only asked when the immediately preceding questions was answered with “yes”. Following questions about their demographic background and dementia, the main body consisted of questions regarding application scenarios of LLMs as well as desired features for digital apps. Finally, participants were asked to estimate the overall impact AI can have on dementia management and care, and there was space for free text with any notes or additions participants would like to make.

**Table 3:** Overview over the questions used in the questionnaire.

**Section**	**ID**	**Item**	**Answer type**
Demographics	1.1	Age	Number
Demographics	1.2	Sex	Multiple choice (Male, Female, Other)
Demographics	1.3	Ethnic background	Multiple choice (Indigenous, Asian, European, African, Pacific Islander, Mixed background)
Demographics	1.4	What is the highest education level you achieved?	Multiple choice (Primary education, Secondary education, Vocational training, BSc, Postgraduate)
Demographics	1.5	Have you ever been diagnosed with dementia or Alzheimer's disease?	Multiple choice (Yes, No)
Demographics	1.6	How many years ago have you been diagnosed with dementia?	Number
Dementia (follow-up)	2.1	What specific type of dementia have you been diagnosed with?	Multiple choice (Alzheimer's, Lewy body dementia, Vascular dementia, Fronto-temporal dementia)
Dementia (follow-up)	2.2	Could you describe any symptoms or experiences related to your diagnosis?	Free text
AI experience	3.1	Have you ever used digital apps in the context of dementia management or treatment?	Multiple choice (Yes, No)
AI experience	3.2	Before starting this questionnaire, had you heard of AI Language Models such as Chat-GPT?	Multiple choice (Yes, No)
AI experience	3.3	Did you ever use AI Language Models such as Chat-GPT (for either personal or professional use)?	Multiple choice (Yes, No)
AI experience (follow-up)	3.4	Please briefly describe how you used AI language models.	Free text
Application scenarios	4.1	**Companionship**. Imagine your app includes a chat option. You can chat with the AI about daily or private matters, questions and concerns. Your conversation is confidential and will not be shared with others. To what extent could you consider such an App useful for yourself?	5-points Likert scale (Very useful, Useful, Moderately useful, Slightly useful, Not useful at all)
Application scenarios	4.2	**Dementia-related information**. Imagine the AI is knowledgeable in the dementia literature. You can ask the AI questions about dementia and you can have a natural conversation in which it provides information about dementia diagnosis, care, treatment etc. *However, it does not have access to your personal medical record, so it can only answer general questions*.	5-points Likert scale
Application scenarios	4.3	**Dementia-related information including personal data**. Imagine the AI is knowledgeable in the dementia literature. You can ask the AI questions about dementia and you can have a natural conversation in which it provides information about dementia diagnosis, care, treatment etc. *The AI also has access to your medical data and it can provide answers tailored to your specific medical conditions*.	5-points Likert scale
Application scenarios	4.4	**Navigation**. Imagine the AI is connected to a navigation system (such as Google Maps or Apple Maps). It can give you directions in spoken language and can help you out if you lose your way.	5-points Likert scale
Application scenarios	4.5	**Reading aid**. Imagine the AI can help you read letters and messages. You simply take a photo of the letter or copy the text into an App. The AI will explain in simple terms what the letter or message means. You can even ask questions about it.	5-points Likert scale
Application scenarios	4.6	**Writing aid**. Imagine the AI can help you draft letters and messages. You simply give it an instruction such as “Write an email to my doctor asking to shift our appointment to next week” and it will give you a nicely written email draft.	5-points Likert scale
Application scenarios	4.7	**Therapy aid**. Imagine the AI is able to carry conversation-based therapeutic interventions such as reminiscence therapy^*^. ^*^Reminiscence therapy involves discussing events and experiences from the past and aims to evoke memories, stimulate mental activity and improve a person's well-being. Reminiscence can often be supported by props such as videos, music, pictures and objects that may have particular meaning for an individual.	5-points Likert scale
Application scenarios	4.8	Do you have any comments regarding these application scenarios? Can you think of any other application scenarios not mentioned here? (feel free to skip this question if 'no')	Free text
Features and priorities	5.1	**Ease of use**. How important is it that the app is intuitive and easy to use, without the need to go through tutorials or receive an introduction by a family member or caregiver?	5-points Likert scale (Very important, Important, Moderately important, Slightly important, Not important)
Features and priorities	5.2	**Voice control**. How important is it that you can also use your voice to talk to the app and it talks back to you (as opposed to just typing text in a textbox)?	5-points Likert scale
Features and priorities	5.3	**Empathy**. When having a conversation with the app, how important is it that the AI displays empathy, feelings, and understanding?	5-points Likert scale
Features and priorities	5.4	**Human in the loop**. When using the app for therapeutic interventions, how important is it to use the Apptogether with in-person sessions with a caregiver or doctor, rather than just using the Appalone?	5-points Likert scale
Features and priorities	5.5	**Data privacy**. How important is it that the app stores as little personal data as possible (e.g., age, gender, past conversations)?	5-points Likert scale
Features and priorities	5.6	**Data transparency**. How important is it that the app is transparent and clear about which data it collects about you?	5-points Likert scale
Features and priorities	5.7	**Data deletion**. How important is it that your personal data can be deleted from the app at any time?	5-points Likert scale
Features and priorities	5.8	**Device**. When using the app, which device(s) do you prefer (select 1 or more)	Multiple choice (Smartphone, Tablet, Laptop or PC)
Conclusion	6.1	**Impact**. What do you estimate the impact of AI on dementia management and care could be?	5-points Likert scale (Very positive, Positive, Neutral, Negative, Very negative)
Conclusion	6.2	**Comments**. If you have any comments, thoughts or suggestions, you can share them with us here.	Free text

The meaning of the columns is as follows. Section: Which section of the questionnaire the question belongs to. ID: identifier of the item that is used in the results section. Item: the verbatim question used in the questionnaire. Answer type: the type of answer that was required, i.e., number (participants entered a number with the keyboard), multiple choice (with the different options provided in brackets), free text (participants type a text as answer), 5-points Likert scale.

#### Procedure

The survey was implemented in Google Forms. It commenced by asking participants to provide informed consent in line with Cardiff University's guidelines. Participants were then asked to watch a 1-min overview over ChatGTP on YouTube (https://www.youtube.com/watch?v=aIO9it4HFiQ) to make sure that they are familiar with the basic principles of LLMs. Further videos and a blog post were presented as optional additional material. They then answered the questions listed in Table 3 by either clicking on the multiple choice options or typing an answer. The survey took about 20 min.

## Results

The raw data and results of the questionnaire are available in our GitHub repository (https://github.com/treder/LLMs-for-dementia). We review the results according to the sections in Table 3: Demographics, dementia, AI experience, application scenarios, and features and priorities.

### Demographics and dementia

**Figure 4:**
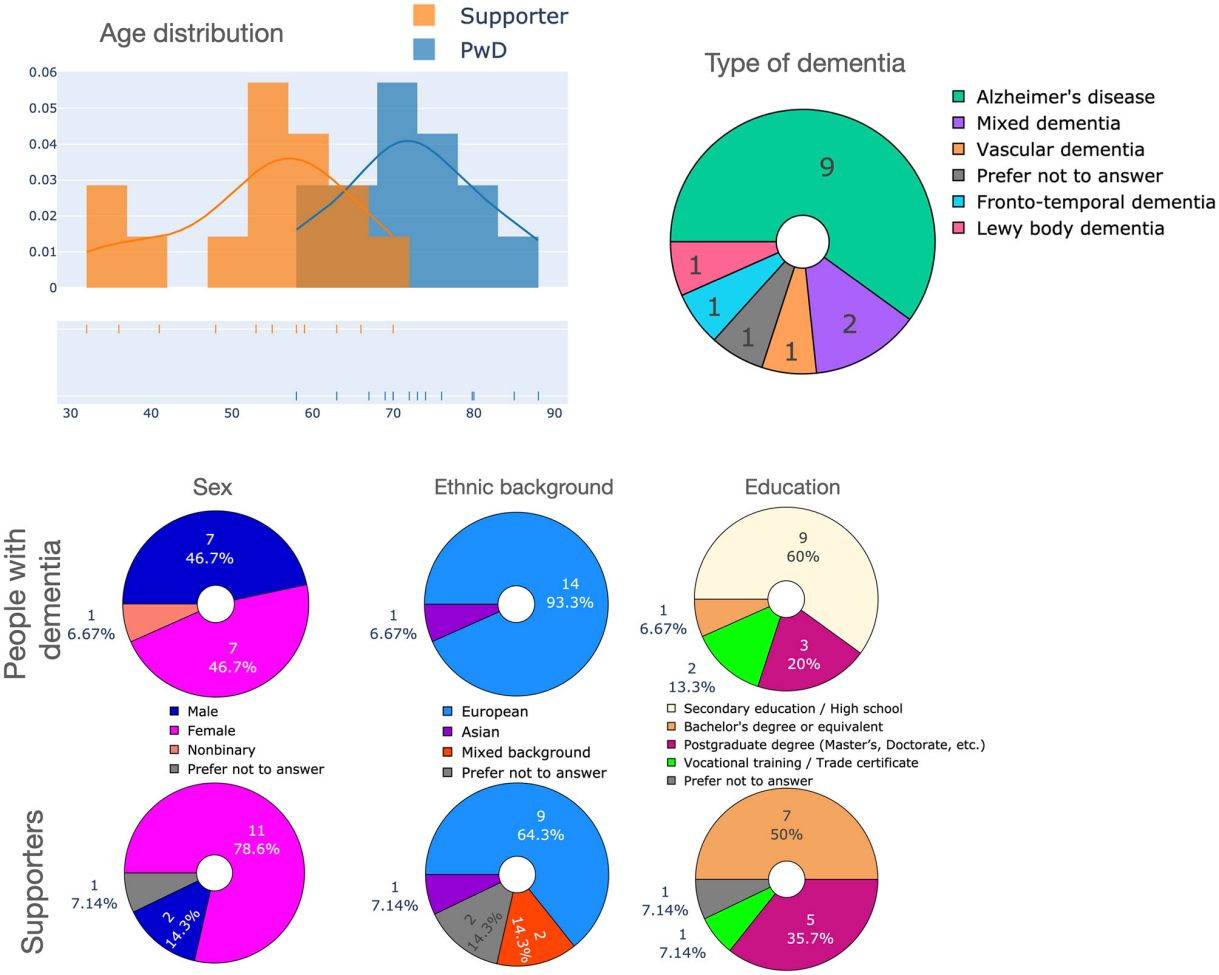
Demographic data of the participants. **Top left**: age distribution for people with dementia (PwD) and supporters. **Top right**: types of dementia. **Bottom row**: sex, ethnic background, and education for people with dementia and supporters.

Figure 4 depicts the demographic details of the participants. People with dementia (PwD) participating in our study were aged 58–88 years whereas supporters were aged 32–70 years. As these ranges suggest, supporters were significantly younger than PwD (independent samples *t*-test, t = 5.059, *p* < 0.0001). Whereas gender roles were equally distributed for PwD (7 women, 7 men, 1 of nonbinary gender), supporters were predominantly female (11 women, 2 men). To compare the distribution of genders across the two groups, we used a two-sided Fisher's exact test which works on 2x2 contingency tables. The chi-squared test allows for larger tables but requires a larger sample size (Hazra and Gogtay, 2016; Sundjaja et al., 2024). Therefore, we focused on comparing the number of men and women. The difference was not statistically significant (odds ratio = 5.5, *p* = 0.1032) which might be attributed to the small sample size.

People with dementia identified as European (14) and Asian (1). Their highest degrees were vocational training/trade certificate (2 respondents), secondary education/high school (9), Bachelor's degree or equivalent (1), or Postgraduate degree (3). The supporters identified as European (9), Mixed background (2), or Asian (1), and 2 preferred not to answer. Supporters had vocational training/trade certificates (1 respondent), Bachelor's degree or equivalent (7), or postgraduate degree (5), and 1 preferred not to answer.

People with dementia received the diagnosis between 1 and 13 years (μ = 5.1) ago. They were diagnosed with various types of dementia, namely Alzheimer's disease (9 respondents), Vascular dementia (1), Fronto-temporal dementia (1), Lewy body dementia (1), or Mixed Dementia (2), and 1 preferred not to answer. When asked to freely describe their symptoms, memory problems were mentioned most often, with 5 respondents mentioned problems with “short term memory”, and another one “total blank in the mornings”. Additional symptoms were related to social interaction (“withdrawn from people”, “unable to speak properly, difficulty understanding conversations”), physical symptoms (“tremors, gait and balance”, “difficult to balance on one side”, “shakes, unstable”), as well as “hallucinations, visual and auditory” and a general “inability to perform everyday tasks” and “inability to understand controls on oven or television”.

### AI experience

Responses related to the use of apps in the context of dementia, 3/15 PwD and 5/14 supporters (1 preferred not to answer) responded with “Yes”. Six PwD and 12 supporters heard of LLMs such as Chat-GPT before. Two PwD (1 preferred not to answer) and 5 supporters (1 preferred not to answer) stated having used them before. One participant with dementia stated that they “use ChatGPT to gather information, links and quotes”. Supporters used them for “Patient and Public Involvement Networks, Universities, and as a carer for my Husband who had Dementia”, to “discover information on various topics encompassing dementia, including the types, symptoms and possible outcomes of therapies used in behavior management in dementia”, as well as to “synthesize text and videos” and for “writing reports”.

### Application scenarios

**Figure 5:**
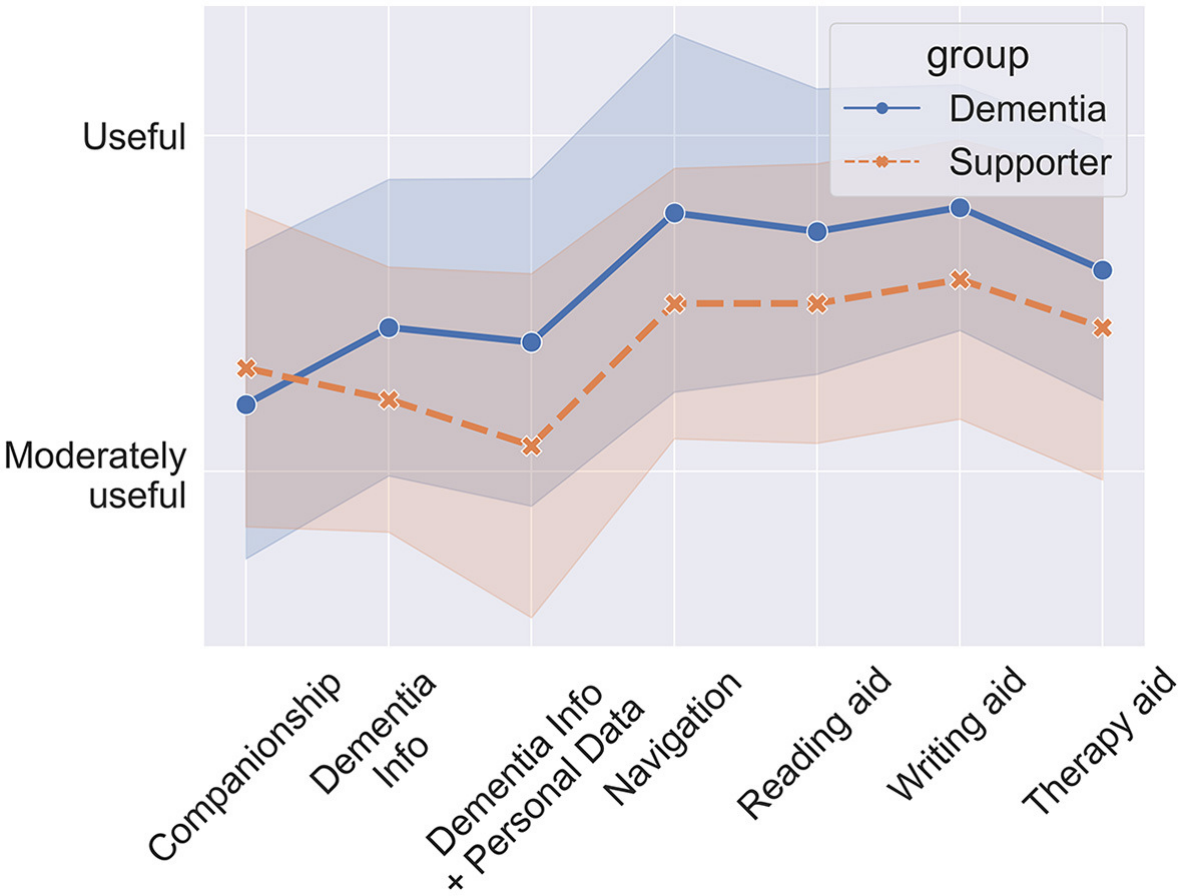
Opinion scores on Likert scale (y-axis) for each of the scenarios (x-axis). Scores have been averaged for individuals within the PwD and supporters groups. Markers depict mean, shaded area represents 1 standard error of the mean.

Figure 5 shows mean opinion scores obtained by encoding the response options as integers ranging from 1 to 5 and averaging them across individuals for the PwD and supporter groups separately. On average, all scenarios were ranked with moderate scores in between “Moderately useful” and “Useful” by both groups. Both PwD and supporters ranked “Navigation”, “Reading aid”, and “Writing aid” the highest. Somewhat lower scores were assigned to “Companionship” and the two items on “Dementia-related information”. As visual inspection suggests, responses between PwD and supporters were significantly correlated (Pearson correlation, *r* = 0.79, *p* = 0.033).

**Figure 6:**
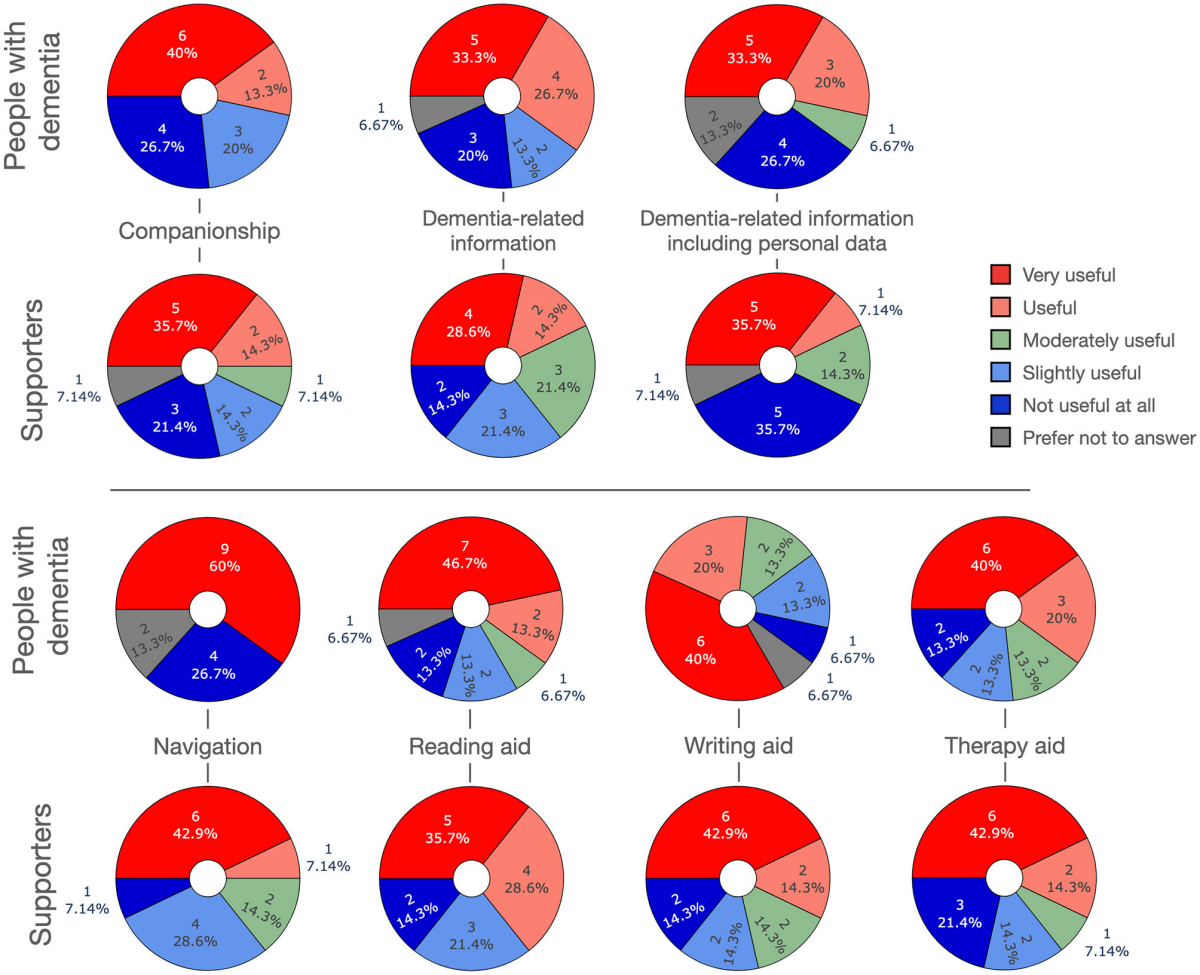
Questionnaire results for seven different application scenarios (see Table 3, IDs 4.1 through 4.7). Pie charts show how useful PwD and supporters consider LLMs in each application scenario. Each scenario is labeled in the figure. The corresponding pie chart is shown for PwD above the label and for supporters below. The numbers in the pie slices correspond to absolute and relative number of respondents (e.g., six respondents, 6/15 = 40%). The legend defines the meaning of the colors.

A more detailed overview with the proportion of each response option by group is depicted in Figure 6. We observe a dichotomy within the PwD group: for each scenario, at least one participant selected the response “Not useful at all” whereas several participants selected “Very useful”. To investigate whether individual response patterns are correlated with demographic variables, we performed a series of Pearson correlation analyses. We found that the overall mean score across all scenarios is negatively correlated with age for PwD (r = −0.62, *p* = 0.014) but not with the number of years since the dementia diagnosis (*p* = 0.42). In other words, older participants tended to give lower overall scores. For supporters, there was no evidence for such a relationship (r = −0.36, *p* = 0.2). When performing the same analysis on the score for each scenario separately, we found a significant relationship for “Companionship” (r = −0.64, *p* = 0.001), “Dementia-related information” (r = −0.54, *p* = 0.044), “Dementia-related information including personal data” (r = −0.61, *p* = 0.027), “Reading aid” (r = −0.61, *p* = 0.019), “Writing aid” (r = −0.62, *p* = 0.002), although only “Companionship” and “Writing aid” would survive a correction for multiple comparisons. Correlations were not significant for “Navigation” (*p* = 0.24). No such relationships were found for supporters (all *p* > 0.16). For the PwD group, we repeated the correlation analysis using the number of years since the dementia diagnosis instead of age, but found no significant effects (all *p* > 0.31). For sex, we did not find a relationship with mean score for either group (all *p* > 0.53).

### Features and priorities

**Figure 7:**
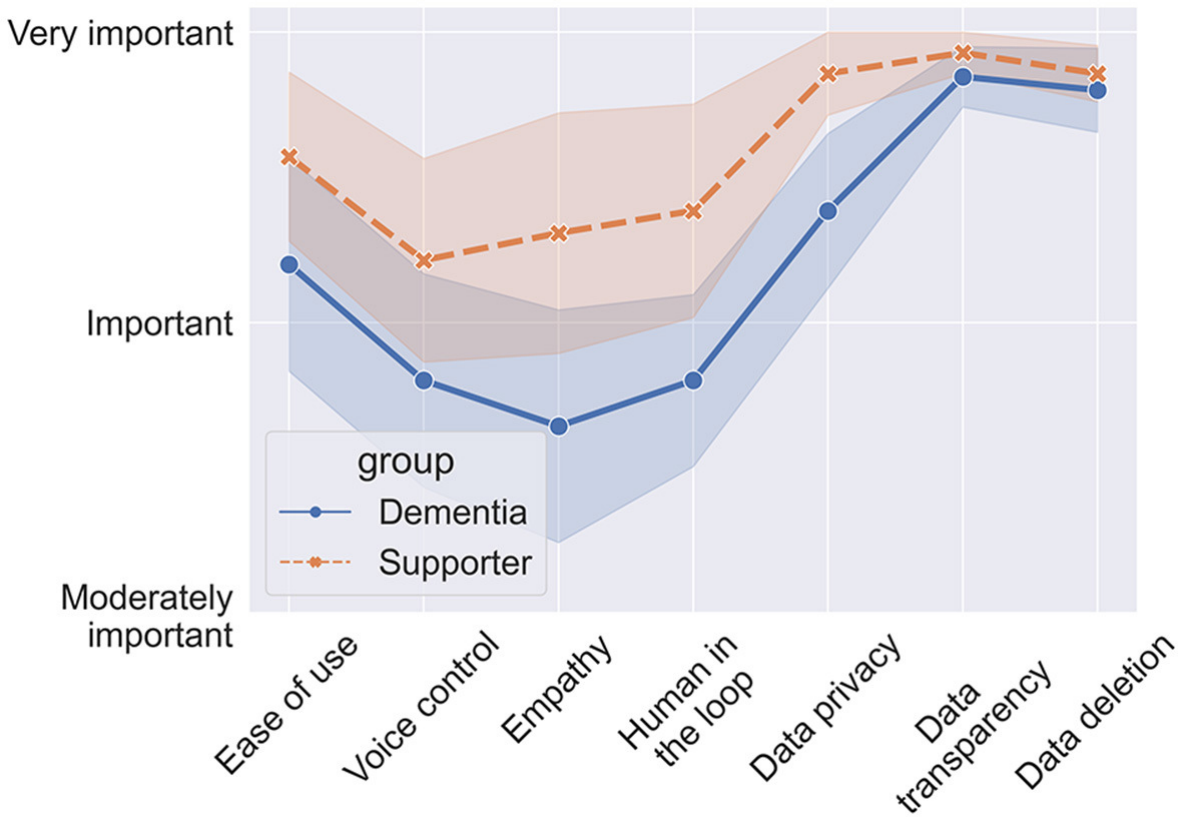
Opinion scores on Likert scale (y-axis) for each of the features and priorities (x-axis). Scores have been averaged for individuals within the PwD and supporters groups. Markers depict mean, shaded area represents 1 standard error of the mean.

Figure 7 shows mean opinion scores obtained by encoding the response options as integers ranging from 1 to 5 and averaging them across individuals for the PwD and supporter groups separately. On average, all scenarios were ranked with moderate to high scores in between “Moderately important” and “Very important” by both groups. Both PwD and supporters ranked all priorities around data (“Data privacy”, “Data transparency”, “Data deletion”) the highest, showing concern for their agency over data. Mean scores between the two groups were highly correlated (Pearson correlation, *r* = 0.94, *p* = 0.001), showing a similar pattern of concerns and priorities.

**Figure 8:**
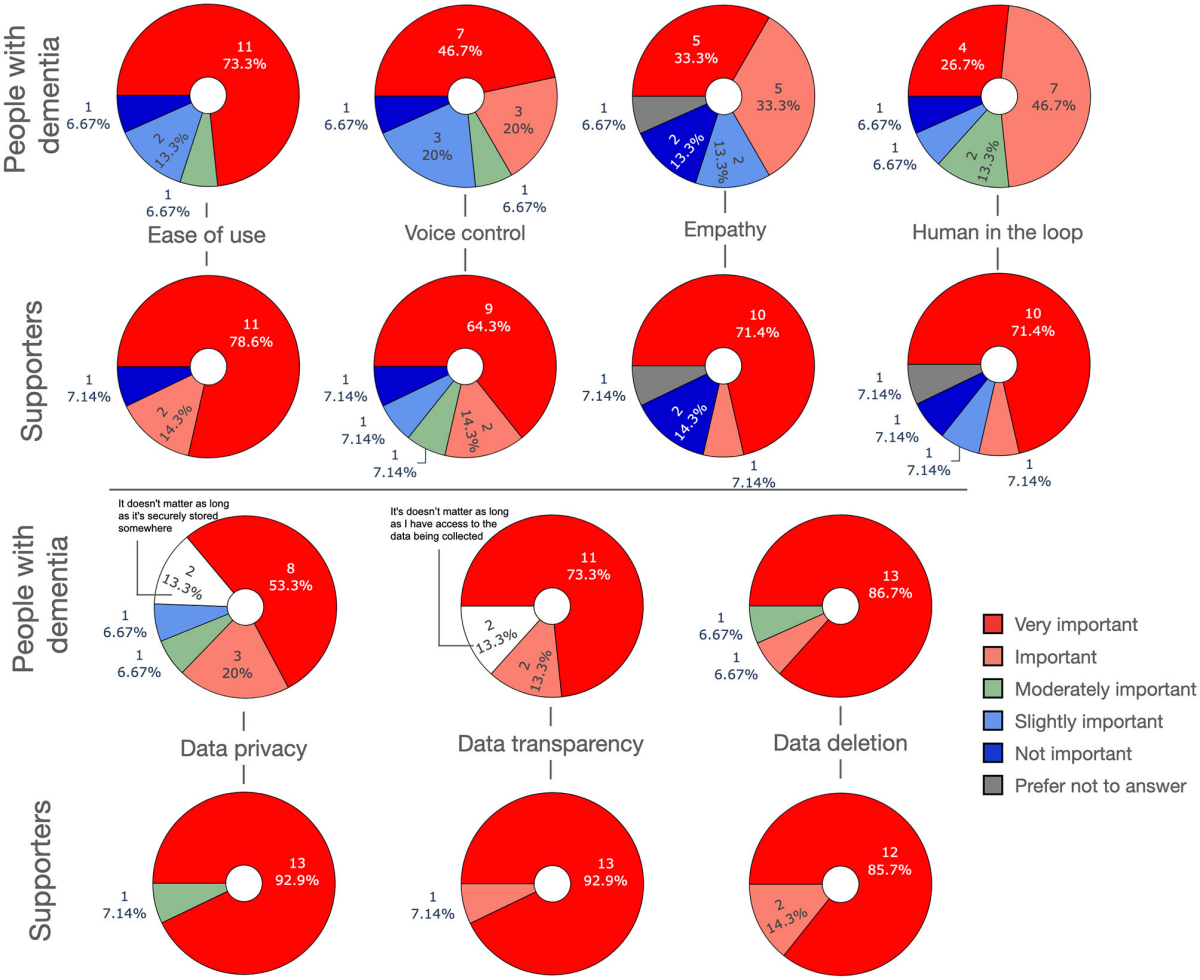
Questionnaire results for features and priorities (see Table 3, IDs 5.1–5.7). Pie charts show how important PwD and supporters consider specific aspects of LLM-based apps for dementia. Each feature is labeled in the figure. The corresponding pie chart is shown for PwD above the label and for supporters below. The numbers in the pie slices correspond to absolute and relative number of respondents (e.g., 11 respondents, 11/15 = 73.3%). The legend defines the meaning of the colors. For data privacy and data transparency, an additional option was provided. The corresponding slice is depicted in white and the response option is pasted next to the figure.

A more detailed overview with the proportion of each response option by group is depicted in Figure 8. Respondents in the PwD group gave either high or low scores to the items “Ease of use”, “Voice control”, “Empathy”, and “Human in the loop”, whereas supporters overwhelmingly gave high scores to these items. Overall mean score across all features and priorities was significantly correlated with age for PwD (r = −0.54, *p* = 0.04) but not supporters (*p* = 0.5). For PwD, when performing the same analyses on the score for each feature and priority separately, we found no significant relationships (all *p*>0.05). There was no significant correlation with sex (PwD: *p* = 0.22, supporters: *p* = 0.17) and for PwD there was no correlation with the number of years since diagnosis (*p* = 0.33).

**Figure 9:**
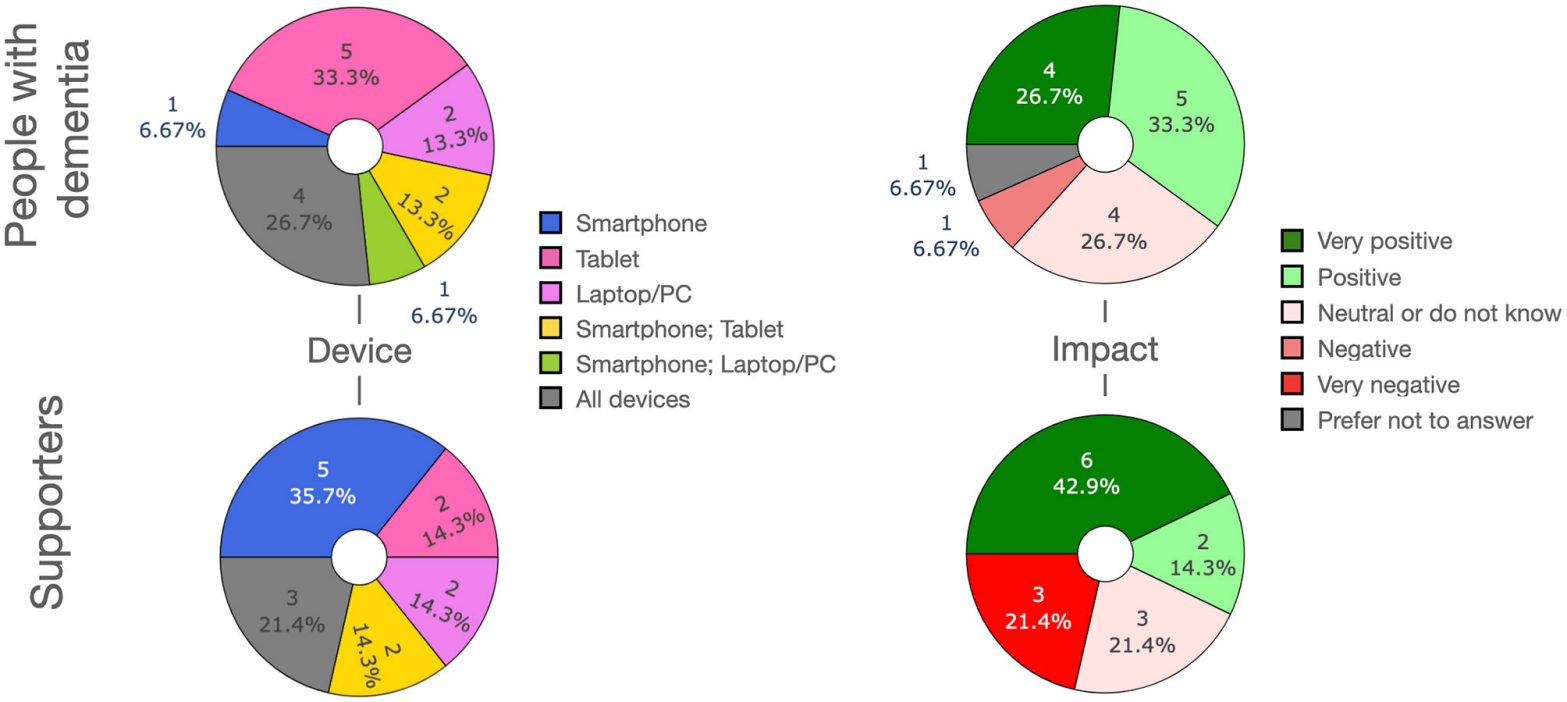
Questionnaire results for device and impact (see Table 3, IDs 5.8 and 6.1). Different legends are provided for each question. For the preferred device, participants could select smartphone, Laptop/PC, tablet, or combinations of these.

Figure 9 depicts results on which devices respondents use and how they rate the overall impact of LLMs on dementia management and care. In both groups a variety of devices was used, although amongst PwD tablets were more dominant whereas among supporters smartphones were more dominant. The overall impact of LLMs on dementia care and management was seen more positively by PwD than supporters. Whereas only 1 respondent in the PwD group indicated “negative”, 3 supporters indicated the impact as “very negative”. Nevertheless, larger proportions in both groups rated the impact as “positive” or “very positive” (PwD: 9 respondents, supporters: 8).

### Free comments

Respondents could also provide feedback in a free textual form (items 4.8 and 6.2 in Table 3). Both PwD and supporters provided feedback on positive and negative use cases for language-based AI applications in dementia. While some respondents were excited about the potential benefits of AI, others raised a number of concerns and caveats. The main points are summarized in Table 4.

**Table 4:** Summary of the feedback of the respondents to the application scenarios.

**Summary**	**Verbatim responses**
The application scenarios are useful	^D^ “*Wow!! I would love anything like those above”* ^D^ “*A product like this would be amazing for me it would take a lot of stress out of my everyday life”* ^S^ “*I think AI has great implications for dementia awareness/care [...]”* ^S^ “*AI technology will be very important to alleviate isolation, and feelings of loneliness for those living with dementia who do not have family or friends nearby to engage with”*
There are other useful application scenarios beyond those mentioned	^D^ “*keeping fit and retaining muscle mass”* ^D^ “*ask medical questions”* ^S^“*Protection from scams would be useful”*
The application scenarios do not address the actual problems of PwD	^D^ “*None of them relate to alleviating the problems of daily living [...]”* ^D^ “*What I really need is something that tells me step by step (all 176 of therm) how to live my daily life”* ^D^ “*I want raw, accurate information that I can easily verify, not a cozy chat. The whole idea of that side of AI is anathema to me”* ^D^ “*As a former carer of someone with Alzheimer's I can see very little use for this app except for navigating IF the person is out alone.”* ^D^ “*My cognitive faculties are relatively intact, it's my recall that is impaired”* ^S^ ”*Tech is NOT the panacea that those who advocate it believe.”*
Concern about bias	^D^ “*dementia care [...] is infected with assumptions of ageism [...] and a host of other biases that are likely to show up in AI development”*
Concern about level of tech affinity required	^D^ “*This AI is not relevant in any way to my mum who has dementia aged 88yrs and has never been able to use a computer even before her diagnosis”* ^D^ “*This AI sounds like amazing progress but the actual demographic of most dementia sufferers is that they are over 70 so I am not too sure AI is going to help them a great deal as they will probably mostly not be computer literate! [...] I am 66 years old and find AI rather a challenge”* ^S^ “*They would find a PC, smartphone etc. very hard to navigate without help“* ^S^ “*it would not be suited to older people with no AI experience. People should start using the planned App as soon as possible so they are familiar with it even before a diagnosis”*
Usefulness depends on stage of dementia	^D^ “*this would be great for FTD and MCI”* ^S^ “*The ability to interact with the AI model depends on the degree of decline”* ^S^ “*might work well very early on in the disease”* ^S^ “*for people with early onset dementia, it could be a valuable tool“* ^S^ “*it just feels unsuitable, totally depends on the stage of dementia, at present it just feels like a gimmick”*
LLM may not understand the user	^S^ ”*If relatives with a good knowledge of the person with other visual cues have difficulty in understanding, it is possible that AI will miss the point.”*
User may not understand the LLM	^S^ “*My clients if having a bad time being lost won't be able follow instructions to find their way home. Also talking about dementia, treatment, diagnosis I can see that leading to confusion, processing that much information, and it might be conflicting information to the client.”*
LLM cannot replace human interaction and care	^S^ “*I know my loved one would not have been happy talking to a machine. It is not a replacement for a human [...] It's too untried to be let loose with those with a dementia diagnosis”* ^S^ “*[...] using the AI app without help from a carer/district nurse or family member would be very difficult for someone in the later stages of dementia”* ^S^ “*It is wrong in so many ways to use a machine to replicate a human response [...] This is very much along the lines of “babysitting by television”*

The first column summarizes the respondents' statements, the second column provides evidence in the shape of the actual individual feedback. The superscript ^D^ refers to respondents in the dementia group and ^S^ refers to supporters.

## Discussion

Large Language Models (LLMs) revolutionize the way in which humans interact with machines. For the first time in history, we can converse with computers in the same way that we talk to each other. Meaningful conversations, creative writing, poetry, summarization, all deeply human faculties that can now be experienced in a chat with an algorithm. Our review has highlighted the burgeoning role of LLMs in improving dementia care and research. The integration of LLMs into therapeutic and support frameworks holds the potential to enhance the quality of life for individuals living with dementia, as well as to alleviate the considerable burden on caregivers. Through personalized conversations, information retrieval, therapy aid, and assistive technologies for reading, writing, and navigation, LLMs offer a novel approach to dementia care that is both innovative and human-centric. Nevertheless, its adoption might face an uphill battle due to algorithmic and regulatory limitations and challenges, as well as concerns about adequacy and applicability in the context of dementia care that surfaced in our survey.

### Limitations, risks, and challenges

Despite the promising prospects, the deployment of LLMs in dementia care is not without challenges. The current limitations of LLMs, including their dependency on the quality of input data and the potential for perpetuating biases, must be acknowledged and addressed. First, hallucinations, or the production of syntactically correct but factually incorrect text, plague all state of the art LLMs (Ye et al., 2023; Zhang et al., 2023) and are a source of concern for their medical application (Pal et al., 2023; Tian et al., 2024). Second, LLMs are trained on a large corpus of text from a variety of sources including social media websites, often without permission of the author of the text (Franceschelli and Musolesi, 2022; Kasneci et al., 2023). Since stigma against people with dementia has been reported on the media platform X (Oscar et al., 2017; Bacsu et al., 2022), inclusion of uncurated internet data into LLM training harbors the danger of perpetuating stereotypes about dementia. Third, overreliance might create adverse cognitive effects. LLMs assisting with perceptual tasks, memory, and language, creates short term benefits, but it is the same faculties that have to be engaged in order to combat decline (Mondini et al., 2016; Hill et al., 2017). Fourth, the development of LLMs for dementia has to take place with a regulatory framework that ensures that risks are mitigated, privacy is preserved, intellectual properties are warranted, and liability for malpractice is established (Meskó and Topol, 2023).

### Questionnaire

Using a questionnaire, we probed both people with dementia (PwD) and their supporters regarding their opinions on the application and features of LLMs in the context of dementia. Participants covered a representative age range for PwD spanning 58–88 years (Hugo and Ganguli, 2014). Whereas the gender split was roughly equal for PwD, the majority of the supporters were women, in line with the predominance of female carers in mental illnesses more broadly (Sharma et al., 2016). Only 3 of 15 Pwd and 5 out of 14 supporters reported ever having used apps in the context of dementia. We presented several application scenarios involving companionship, dementia information, navigation, reading or writing aid, and therapy aid. Both PwD and supporters rated all of the scenarios as moderately useful to useful. Older PwD tended to give lower overall scores than younger PwD. It is up to speculation as to why, perhaps indicating a generally more negative outlook, the existence of more severe symptoms that are unlikely to be alleviated by LLMs, or perhaps a larger barrier to use digital apps.

Regarding their priorities for what features LLM-powered apps should have, PwD and supporters ranked agency over data (privacy, transparency, deletion) and ease of use the highest. Opinions on the usefulness of the technology diverged, however. In the free comments sections (see Table 4), some respondents praised the promise of the technology (“I think AI has great implications for dementia awareness/care [...]”) but they also raised several caveats. The application scenarios might not address the real needs of PwD (“None of them relate to alleviating the problems of daily living [...]”). There were also concerns about bias and technological affinity required (“This AI sounds like amazing progress but the actual demographic of most dementia sufferers is that they are over 70 so i am not too sure AI is going to help them a great deal as they will probably mostly not be computer literate”). Other respondents pointed out that the usefulness of LLMs depends on the stage of dementia (“The ability to interact with the AI model depends on the degree of decline”) and that LLMs cannot serve as a substitute for human interaction (“I know my loved one would not have been happy talking to a machine. It is not a replacement for a human”).

### Limitations of the questionnaire

Our online survey has several limitations. The use of convenience sampling through dementia organizations as gatekeepers, while practical, may introduce selection bias. This approach relies on participants who are actively engaged with these organizations and have access to the internet, potentially excluding a portion of the dementia population who are less active or lack online access. Additionally, sample sizes of 15 people with dementia and 14 supporters limit the statistical power of the study especially with regard to more subtle effects. It is also possible that in some cases both a PwD and their supporter filled in the questionnaire, leading to correlation between the samples. The anonymity of the questionnaire, while protecting participant privacy and potentially lowering the barrier to participation, also prevents any follow-up for more in-depth data collection.

### Implications for practice

Our findings suggest that LLMs can serve as an invaluable resource in dementia care. By providing personalized interaction and support, LLMs have the potential to improve social engagement and cognitive functioning among individuals with dementia. However, the successful implementation of LLMs in dementia care requires careful consideration of the technology's limitations and the ethical implications of its deployment. Privacy and safety concerns must be meticulously addressed, and systems need to be designed with the end-user in mind, ensuring that they are accessible, intuitive, and genuinely beneficial.

When our previous considerations and the findings from the survey are taken together, we can add the following points for LLM-powered apps for dementia care and management:

- **On-demand aid**. LLM-powered apps offer on-demand and non-judgmental support, potentially including mental health benefits such as promotion of self-confidence and aiding self-discovery (Ma et al., 2024).
- **Caregiver burden**. Economics mandate a reduction in care cost (Nandi et al., 2022). While LLMs might alleviate caregiver burden, many respondents pointed out that apps cannot serve as substitutes to human interaction. Most likely, a collaborative solution involving human support augmented by a chatbot for periods wherein the human supporter is not available would be a way forward that meets targets both in terms of quality of care and associated monetary cost.
- **Prompt engineering and communication**. It is yet unclear how LLMs perform in the presence of language disorder (Murdoch et al., 1987) and other forms of cognitive impairment (Hugo and Ganguli, 2014). It is conceivable that bespoke models are required, e.g. by finetuning a model such as ChatGPT on a dataset including excerpts of speech from language-impaired individuals. Since the communication is bi-directional, further finetuning might be required to align the model to produce outputs that are more palatable for individuals with impairments.
- **Complexity and technological affinity**. As long as operating LLMs is not seamless any real-world implementation faces a catch-22 scenario: users that benefit from an LLM the most might find it the most challenging to operate LLM-powered apps. For this reason, some respondents pointed out that LLMs should be aimed toward milder versions of dementia such as early-onset dementia. Integration in physical agents such as robots could provide a more seamless gateway between LLM and the user.
- **Co-development of apps**. As a note to tech developers, our survey showed the importance of co-developing solutions with the end user (both PwD and supporters) in the loop early. Otherwise one runs the risk of designing a solution that does not address the needs of PwD or is not usable in the light of their expertise and challenges in using such apps. Furthermore, the language used should not patronize PwD or diminish their agency or cognitive capacities.
- **Data agency**. PwD and supporters stressed the importance of retaining agency over their digital data, including transparency about its usage and the ability to delete it.

### Future research directions

To harness the full potential of LLMs in dementia care, future research should focus on several key areas. First, there is a need for longitudinal studies to assess the long-term impact of LLM interactions on individuals with dementia. This includes evaluating the effects on cognitive health, emotional wellbeing, and social engagement over time. It also involves a better characterization of dementia-specific limitations and risks associated with the usage of LLMs. Second, research should explore the customization and personalization of LLMs to meet the diverse needs of individuals with dementia. This includes the development of adaptive algorithms that can tailor interactions based on the user's preferences, behaviors, and cognitive status. Third, the exploration of multimodal LLMs that can interpret and respond to non-verbal cues could significantly enhance the quality of interactions, making the technology more accessible and effective for individuals with varying degrees of cognitive impairment. Fourth, exploring the embodiment of LLMs in robotics could revolutionize dementia care by providing conversational and physical support through social robots (D'Onofrio et al., 2019; Fields et al., 2021; Lima et al., 2022). This research should aim to develop adaptive robots that cater to the emotional and physical needs of dementia patients, enhancing their quality of life with a blend of cognitive support and companionship.

### Conclusion

In conclusion, the use of Large Language Models in dementia care represents a promising frontier in the intersection of AI and healthcare. While challenges and limitations exist, the potential benefits of LLMs in enhancing cognitive abilities, enriching social interaction, and supporting caregivers are undeniable. As we move forward, it is crucial that the development and implementation of LLMs is guided by ethical considerations, empirical evidence, and a commitment to improving the lives of individuals living with dementia.

## Data availability statement

The raw data and a Jupyter notebook reproducing the results of the questionnaire are available in our GitHub repository (https://github.com/treder/LLMs-for-dementia).

## Ethics statement

The studies involving humans were approved by the School of Computer Science and Informatics Research Ethics Committee at Cardiff University, United Kingdom (reference code COMSC/Ethics/2023/122). The studies were conducted in accordance with the local legislation and institutional requirements. The participants provided their written informed consent to participate in this study.

## Author contributions

MT: Conceptualization, Data curation, Investigation, Formal analysis, Methodology, Visualization, Writing – review & editing, Writing – original draft. SL: Investigation, Writing – review & editing, Writing – original draft. KT: Writing – review & editing, Writing – original draft.
