# Supplementary material for: Introduction to Large Language Models (LLMs) for dementia care and research
Source: Front Dement. 2024 May 14;3:1385303. doi: 10.3389/frdem.2024.1385303 (PMC11285660; doi:10.3389/frdem.2024.1385303)
Supplement: Supplementary file 1 [file Data_Sheet_1.docx]

Supplemental Materials

## Supplement A. Dementia overview

### Epidemiology

Dementia is a public health priority (Prince et al., 2015). The number of people with dementia is expected to triple by 2050 (Nichols et al., 2022). This surge presents a huge burden on health and social care, with one in three people expected to experience dementia onset. The economic impact is staggering, with costs projected to escalate from $2.8 trillion annually in 2019 to $4.7 trillion by 2030 and $16.9 trillion in 2050 (Nandi et al., 2022). Informal care accounts for nearly 50% of costs in high-income countries, but this proportion is even higher for low- and middle-income countries where social care covers only 15% (Prince et al., 2015). These projections underscore the urgent need for innovative, scalable solutions and care modes to address personal, clinical and economic consequences of dementia globally.

### Classification and characteristics of dementia syndromes

Dementia is marked by a gradual decline in cognitive and motor functions resulting from the progressive loss of brain cells. The symptoms typically occur in a continuum over several stages. This deterioration severely impacts behaviour, emotions, relationships and daily functioning. Dementia is not a single disease, but an umbrella term used to describe various brain disorders. The most common types are Alzheimer’s disease, Vascular dementia, dementia with Lewy bodies, frontotemporal dementia. Each type involves damage to particular brain cells in specific regions of the brain, leading to a spectrum of heterogenous symptoms. It is possible to have brain changes associated with more than one type of dementia at the same time, called *mixed* dementia (Kapasi et al., 2017; Schneider et al., 2007). Further complexity arises as these dementia types are further subdivided into genetic or familial categories, as well as those that are sporadic in nature, adding to the nuanced understanding and challenges associated with these disorders.

Alzheimer’s disease is the most prevalent cause of dementia, contributing to 50-75% of cases. Key symptoms are memory lapses, word-finding difficulties, and mood swings (Armstrong et al., 2024; Jack et al., 2018; Sheehan, 2012). Elevated levels of certain proteins inside and outside brain cells interfere with communication between cells and their health leading to the symptoms of Alzheimer’s (Lane et al., 2018). The hippocampus, the brain region responsible for learning and memory, and its brain cells are often the first site of damage. While most Alzheimer’s cases occur sporadically or in a late-onset basis, a rare familial form (<5%) linked to mutations in three genes – amyloid precursor protein, presenilin 1 and presenilin 2 – often presents under the age of 65, known as early-onset (“2023 Alzheimer’s disease facts and figures,” 2023).

Vascular dementia is the second most common cause of dementia, contributing to most of the mixed dementia(Bir et al., 2021; Iadecola et al., 2019). Vascular pathology often co-occurs with Alzheimer’s pathology, synergistically contributing to cognitive impairment (Chun et al., 2024). About 5-10% of dementia are vascular dementia alone (Brenowitz et al., 2017; Kapasi et al., 2017). It occurs due to brain’s blood vessel damage and reduced blood flow to the brain. The location and extent of brain damage depends on which blood vessels are blocked or damaged. Symptoms often include impaired judgment, planning, and organizational skills. Changes in personality and mood fluctuations also frequently occur.

Dementia with Lewy bodies involves abnormal protein deposits called Lewy bodies forming inside brain cells. About 5% dementia cases are dementia Lewy bodies alone, but most Lewy bodies affected brains show comorbid Alzheimer’s pathology (Kane et al., 2018). Early symptoms can include visual hallucinations, Parkinson's-like movement problems, and sleep disturbances. Memory loss and attention fluctuations tend to emerge later. Dementia with Lewy bodies shares some overlap with Alzheimer's and Parkinson's diseases.

Frontotemporal dementia (FTD) causes a spectrum of heterogenous clinical syndromes characterised by the progressive neuron degeneration in the brain's frontal and temporal lobes (Ferrari et al., 2019; Olney et al., 2017). Subcortical involvement may be an important aspect for understanding cognitive sequalae (Bocchetta et al., 2021). FTD differs from other dementias in two major ways. First, FTD is a common cause of young-onset dementia, with approximately 60% of patients being between 45 to 60 years old. Second, FTD impacts different aspects of cognition, personality and behaviour (Coyle-Gilchrist et al., 2016). FTD patients are typically diagnosed as having one of several principal syndromes including behavioural variant (bvFTD), primary progressive aphasias (PPA), with semantic variant (svPPA), nonfluent agrammatic variant (nfvPPA) and logopenic variant; and motor presentations, such as bvFTD with motor neuron disease (MDN) or atypical parkinsonian disorders like progressive supranuclear palsy (PSP, Steele-Richardson-Olszewski syndrome) (Rowe et al., 2021) and corticobasal syndrome (Jabbari et al., 2020). More recently a transdiagnostic approach has been proposed, demonstrating that FTD syndromes exist along a multidimensional spectrum rather than as discrete entities (Murley et al., 2020).

### Diagnosis and neurodegenerative continuum

The diagnosis of dementia typically involves a comprehensive assessment process following consensus-based best practices (Alexander et al., 2014; Armstrong et al., 2024; Gorno-Tempini et al., 2011; Ismail et al., 2020; Rascovsky et al., 2007). A diagnosis of dementia requires observing a deviation from an individual’s normal mental functioning accompanied by a greater cognitive decline than what is expected from normal ageing. In other words, while dementia risk raises with age, it remains distinct from the normal ageing process. The neurodegenerative disease continuum manifest through subtle, initially unnoticeable brain changes in the affected individual, that eventually lead to cognitive impairments and ultimately, physical disability. This continuum includes two pre-dementia stages – preclinical dementia exhibiting unimpaired cognition and a prodromal stage exemplified by mild cognitive impairment in Alzheimer’s disease context – followed by three dementia stages of increased severity: mild, moderate and severe (“2023 Alzheimer’s disease facts and figures,” 2023).

This continuum of progressive neurocognitive decline can be assessed clinically using standardized scales of overall dementia severity, such as the Global Deterioration Scale, the Functional Assessment Staging Test or the Clinical Dementia Rating (Sheehan, 2012). Nevertheless, within the research setting, it is important to consider diverse aspects of the clinical manifestation of dementia. This can complemented with assessment scales of the clinical consequences of the disease (i.e. symptoms/sings) (Sheehan, 2012) and biological markers such as amyloid beta deposition, pathological tau, neurodegeneration (ANT) (Jack et al., 2018), and recently, vascular burden in Alzheimer’s context (Chun et al., 2024).

Assessment scales of the clinical syndrome can focus on screening for cognitive impairment based on professional-patient interaction such as Mini-Mental State Examination (Folstein et al., 1975), Montreal Cognitive Assessment (Nasreddine et al., 2005), and Addenbrooke’s Cognitive Assessment (Mioshi et al., 2006). Assessments can also focus on specific cognitive functions, such as memory (Buschke, 1984; Poos et al., 2021) and language (Peterson et al., 2021). Furthermore, informant-based, rather than patient-based, questionnaires such as the Cambridge Behavioural Inventory (CBI-R) have also been shown to distinguish between dementia subtypes (Wear et al., 2008).

Adhering to these consensus-based best practices assists in making an accurate and timely diagnosis of dementia and enables suitable management and support for patients and their families.

### Risk factors and prevention

The majority of people affected by dementia are age 65 or older, a condition known as late-onset dementia. Dementia is believed to develop as a result of combination of multiple factors rather than a single cause, with age, genetics and family history being the most prominent risk factors (“2023 Alzheimer’s disease facts and figures,” 2023).

Age is the strongest risk factor, with the chances of developing dementia rising dramatically with age. Genetics also play a major role, particularly the presence of APOE e4 gene variant. Individuals carrying one copy of the e4 variant have 3 times the risk of developing Alzheimer’s disease, while those with two copies bear an 8-12 fold increased risk compared to those with copies of the neutral e3 variant. Over half of those diagnosed with Alzheimer’s have at least one copy of the e4 variant.

While age, genetics and family history cannot be changed, some risk factors can be modified to reduce dementia risk. Examples include cardiovascular health, physical activity, smoking, alcohol, education level, hearing loss, social/cognitive engagement, obesity and traumatic brain injury. Addressing 12 modifiable risk factors may prevent or delay up to 40% of dementia cases (Livingston et al., 2020). Importantly, these risk factors are interconnected across the lifespan, with early-life influences impacting outcomes in middle age. Both individual behavioral changes and public health policies can modify these factors. As discussed below, integration of LLM solutions for personalised management and support, extending beyond disease modeling, risk assessment, intervention planning and monitoring (Agbavor and Liang, 2022; Bzdok et al., 2024; de Arriba-Pérez et al., 2023; Demszky et al., 2023; Loconte et al., 2023; Tian et al., 2024; Wang et al., 2023), may further enhance the effectiveness of dementia treatment and prevention strategies.

In summary, while age, genetics, and family history represent the primary risk factors for dementia and remain beyond individual control, focusing on modifiable risk factors, particularly during midlife, may significantly reduce one’s chances of developing Alzheimer’s and other dementias in later years.

### Treatment

Currently, there is no cure for dementia. However, with the right care and support, it is possible for someone to live as well as possible for as long as possible. Various treatments for dementia include pharmacological and non-pharmacological approaches, as well as management strategies.

Conventional pharmacological treatments primarily target symptom improvement rather than disease modification. In other words, no drugs can stop or reverse dementia, only temporarily help manage symptoms. However, recent years have seen significant advancements in dementia treatments, as highlighted by Alzforum’s analysis (https://www.alzforum.org/news/community-news/2023-bumper-year-treatment-and-science-alzheimers). In 2023, the US Food and Drug Administration (FDA) approved several Alzheimer’s drugs, making progress over 2022. Other countries like Japan and China followed. Among these was lecanemab, an anti-amyloid antibody aiming to alter disease biology by removing beta-amyloid and slowing cognitive decline in early-stage Alzheimer’s (Gandy, 2023). However, these drugs have common side effects including headaches, infusion reactions and amyloid-related imaging abnormalities (ARIA) (Honig et al., 2023). Thus, beside developments on improving efficacy of disease-modifying treatments, ongoing efforts also focus on solutions designed to mitigate adverse events associated with ARIA (Doran and Sawyer, 2024).

Finally, the combination of traditional pharmaceutical treatments and non-pharmacological interventions, including physical activity and cognitive exercises, may offer a more effective approach to maintain cognitive function and enhance overall quality of life (Chalfont et al., 2020; Dimitriou et al., 2022; Zucchella et al., 2018). This could be further complemented by proactive management of care strategies, including treatment optimisation, caregiver training, and community support networks, to improve patient outcomes and enhance caregiver well being.

## Supplement B. Brief history of Large Language Models (LLMs)

While LLMs gained widespread popularity in recent years, they are firmly rooted in an area of AI research known as Natural Language Processing (NLP), an interdisciplinary fusion of computer science and linguistics. NLP is primarily concerned with enabling machines to interpret, generate, and respond to human language in a manner akin to our own cognitive abilities. LLMs are the latest iteration towards establishing such machines. Historically, the seeds of NLP can be traced back to the 1950s, with pioneering experiments such as the Georgetown-IBM experiment, which sought to automatically translate Russian sentences into English (Hutchins, 2004). Early NLP endeavors were heavily rule-based, relying on meticulously crafted grammars and lexicons (Basili et al., 1996; Chan and Franklin, 1998), which, while laudable, were often brittle and lacked scalability (Lenat et al., 1985). The late 1980s and 1990s ushered in a paradigm shift, with statistical methods and probabilistic models, especially those predicated on Markov chains and Hidden Markov Models, gaining prominence (Collins, 2002; Fine et al., 1998). This era was characterized by the machine's ability to learn from vast amounts of textual data, rather than relying solely on hardcoded linguistic rules. The subsequent advent of neural networks and deep learning in the 2010s provided an additional impetus to NLP's evolution. Techniques like word embeddings (e.g., word2vec), which offered a dense vector representation of words capturing semantic meaning, became foundational (Goldberg, 2017). Recurrent Neural Networks (RNNs) and their variants like Long Short-Term Memory (LSTM) (Hochreiter and Schmidhuber, 1997) further advanced sequence-based tasks such as translation and sentiment analysis (Minaee et al., 2019; Wu et al., 2016). However, it was the introduction of the transformer architecture in 2017 (Vaswani et al., 2017), predicated on self-attention mechanisms (Parikh et al., 2016), that truly revolutionized the field, enabling the development of high-capacity models like BERT (Devlin et al., 2019) and GPT (Radford et al., 2018) that have since set numerous benchmarks.

## Supplement C. Regulatory challenges

The use of Large Language Models (LLMs) in healthcare holds great opportunities for improving patient outcomes, yet it also presents substantial challenges and risks that must be properly regulated (Meskó and Topol, 2023). Researchers are rightly concerned that, although this technology has transformative potential, its societal risks may outweigh its benefits if not implemented responsibly (Gabriel, 2020). Concerns have been raised, particularly regarding ethics (Floridi and Floridi, 2023; Tsarapatsanis and Aletras, 2021), bias (Blodgett et al., 2020; Hovy and Prabhumoye, 2021), safety (Dobbe et al., 2021), and environmental impact (Etzioni, n.d.; Rillig et al., 2023; Strubell et al., 2019). Proactive regulatory measures are vital to harness the potential benefits of AI-driven technologies like LLMs while mitigating associated risks and maintaining the trust of both patients and healthcare providers. Moreover, LLMs have the potential to be the pioneering category of AI-based medical technologies subject to regulation designed with patient input, ensuring that these rapidly evolving AI tools are tailored to meet real-life clinical and patient needs (Meskó and Topol, 2023).

#### Data Privacy

The potential violation of contextual integrity in the deployment of generative AI models, particularly when trained on personal data taken out of its original context, raises significant privacy concerns for individual consumers. This issue is exemplified by scenarios where individuals unknowingly contribute their personal data, to train AI models without informed consent. As public awareness grows regarding the use of personal data for training generative AI models, it may lead to chilling effects unless existing legislation, such as the general data protection regulation (GDPR), is enforced against companies deploying these models (Norwegian Consumer Council, 2023). Ensuring patient data privacy is a critical consideration when implementing LLMs in healthcare, necessitating stringent regulations for the thorough anonymisation and protection of patient data used in model training to prevent potential breaches. Compliance with patient data privacy laws, such as the Health Insurance Portability and Accountability Act (HIPAA) in the United States, is imperative to avoid severe consequences, highlighting the regulatory challenges faced in deploying LLMs in healthcare settings (Meskó and Topol, 2023). Additionally, the integration of AI tools into healthcare management requires securing informed consent from patients, a complex task due to the challenges patients face in understanding the implications of AI usage. Addressing this complexity within regulatory frameworks is essential to ensure effective patient consent in the context of AI applications in healthcare (Meskó and Topol, 2023).

#### Interpretability and Transparency

Transparency is crucial in areas such as data collection, content moderation decisions, and understanding environmental and social impacts. Unfortunately, some AI developers, including Google, Microsoft, and OpenAI, exhibit a trend of limiting external scrutiny. Concerns arise as closed systems hinder verification and dispute of AI capabilities. Trade agreements may restrict lawmakers from demanding transparency, undermining democratic principles. The lack of transparency extends to service providers using third-party generative AI models, posing challenges in assigning liability and accountability. Collaboration between companies, like Klarna and OpenAI, emphasizes the need for accessible information to evaluate AI system impacts on consumers and enforce regulations effectively (Norwegian Consumer Council, 2023). Regulations must enforce transparency in understanding how AI models make decisions, particularly concerning AI models often described as "black boxes" due to their intricate algorithms (Meskó and Topol, 2023).

#### Fairness and Mitigating Bias

Recent years have seen a growing focus in computer science literature on the risks associated with LLMs. Two main strands of literature have emerged: one relating to fairness and machine learning in general (Barocas et al., 2023) as well as specific examples of algorithmic bias risks in NLP (Field et al., 2023; Kidd and Birhane, 2023; Talat et al., 2022), computer vision (Wolfe et al., 2023), multimodal models (Birhane et al., 2021), as well as privacy risks (Mireshghallah et al., 2022); the other taking a holistic view of LLMs, considering broader risks such as environmental impact, bias, representation, and hate speech (Goanta et al., 2023). Risk and uncertainty frameworks, foundational in risk regulation, involve quantifying risk for policy-making, a complex task, particularly for globally transcendent technologies like LLMs, given their complexity, information asymmetry, and alignment controversies, contributing to

considerable uncertainty (Goanta et al., 2023). Regulatory safeguards are crucial to prevent biases in AI models, which can be inadvertently introduced during the training process using patient data and lead to healthcare disparities (Meskó and Topol, 2023).

#### Liability for Medical Malpractice

Determining liability in cases where AI-generated recommendations result in patient harm presents a regulatory challenge. Regulations need to clarify who bears responsibility: the AI developers, the healthcare professionals implementing the AI, or the institutions adopting it (Meskó and Topol, 2023). The European Commission has introduced a proposal for an updated product liability directive (revised PLD). The updated directive is also meant to cover software, including AI systems (Buscke, 2023). The European Commission has proposed the AI Liability Directive (AILD) alongside the Artificial Intelligence Act (AIA) and as an extension of the Product Liability Directive (PLD), aiming to facilitate consumer compensation for AI-induced harms. While the AILD, currently in draft form, awaits finalization contingent on AIA adoption, it allows consumers to claim compensation for both material and non-material harms, subject to national legal frameworks. However, significant limitations demand consumers prove fault on the part of AI system operators, requiring technical expertise beyond regular capacity. To enhance consumer protection, recommendations include non-fault-based liability and a reversal of the burden of proof. Uncertainties persist on the classification of generative AI under the AIA, potentially making AILD applicable. Yet, compensation claims for inaccuracies from generative AI lack harmonization, necessitating case-specific assessments (Norwegian Consumer Council, 2023).

#### Continuous Monitoring and Validation: Ensuring Reliability Over Time

The continuous performance, accuracy, and validation of AI tools over time and across different populations is a crucial regulatory challenge. Regulatory bodies must establish mechanisms for ongoing monitoring and validation to ensure the effectiveness and safety of AI tools in healthcare scenarios (Meskó and Topol, 2023).

# References

2023 Alzheimer’s disease facts and figures, 2023. . Alzheimers Dement. J. Alzheimers Assoc. 19, 1598–1695. https://doi.org/10.1002/alz.13016

Agbavor, F., Liang, H., 2022. Predicting dementia from spontaneous speech using large language models. PLOS Digit. Health 1, e0000168. https://doi.org/10.1371/journal.pdig.0000168

Alexander, S.K., Rittman, T., Xuereb, J.H., Bak, T.H., Hodges, J.R., Rowe, J.B., 2014. Validation of the new consensus criteria for the diagnosis of corticobasal degeneration. J. Neurol. Neurosurg. Psychiatry 85, 925–929. https://doi.org/10.1136/jnnp-2013-307035

Armstrong, M.J., Bedenfield, N., Rosselli, M., Curiel Cid, R.E., Kitaigorodsky, M., Galvin, J.E., Lachner, C., Grant Smith, A., de los Ángeles Ortega, M., Mohiuddin, Y., Shatzer, J., Marasco, D., Willis, D., Bylund, C.L., 2024. Best Practices for Communicating a Diagnosis of Dementia. Neurol. Clin. Pract. 14, e200223. https://doi.org/10.1212/CPJ.0000000000200223

Barocas, S., Hardt, M., Narayanan, A., 2023. Fairness and Machine Learning. MIT Press.

Basili, R., Pazienza, M.T., Velardi, P., 1996. An empirical symbolic approach to natural language processing. Artif. Intell. 85, 59–99. https://doi.org/10.1016/0004-3702(95)00116-6

Bir, S.C., Khan, M.W., Javalkar, V., Toledo, E.G., Kelley, R.E., 2021. Emerging Concepts in Vascular Dementia: A Review. J. Stroke Cerebrovasc. Dis. Off. J. Natl. Stroke Assoc. 30, 105864. https://doi.org/10.1016/j.jstrokecerebrovasdis.2021.105864

Birhane, A., Prabhu, V.U., Kahembwe, E., 2021. Multimodal datasets: misogyny, pornography, and malignant stereotypes. https://doi.org/10.48550/arXiv.2110.01963

Blodgett, S.L., Barocas, S., Daumé III, H., Wallach, H., 2020. Language (Technology) is Power: A Critical Survey of “Bias” in NLP, in: Jurafsky, D., Chai, J., Schluter, N., Tetreault, J. (Eds.), Proceedings of the 58th Annual Meeting of the Association for Computational Linguistics. Presented at the ACL 2020, Association for Computational Linguistics, Online, pp. 5454–5476. https://doi.org/10.18653/v1/2020.acl-main.485

Bocchetta, M., Malpetti, M., Todd, E.G., Rowe, J.B., Rohrer, J.D., 2021. Looking beneath the surface: the importance of subcortical structures in frontotemporal dementia. Brain Commun. 3, fcab158. https://doi.org/10.1093/braincomms/fcab158

Brenowitz, W.D., Keene, C.D., Hawes, S.E., Hubbard, R.A., Longstreth, W.T., Woltjer, R.L., Crane, P.K., Larson, E.B., Kukull, W.A., 2017. Alzheimer’s disease neuropathologic change, Lewy body disease, and vascular brain injury in clinic- and community-based samples. Neurobiol. Aging 53, 83–92. https://doi.org/10.1016/j.neurobiolaging.2017.01.017

Buschke, H., 1984. Cued recall in amnesia. J. Clin. Neuropsychol. 6, 433–440. https://doi.org/10.1080/01688638408401233

Buscke, U., 2023. Revision of the product liability directive (No. BEUC-X-2023-023), BEUC-The European Consumer Organisation.

Bzdok, D., Thieme, A., Levkovskyy, O., Wren, P., Ray, T., Reddy, S., 2024. Data science opportunities of large language models for neuroscience and biomedicine. Neuron. https://doi.org/10.1016/j.neuron.2024.01.016

Chalfont, G., Milligan, C., Simpson, J., 2020. A mixed methods systematic review of multimodal non-pharmacological interventions to improve cognition for people with dementia. Dementia 19, 1086–1130. https://doi.org/10.1177/1471301218795289

Chan, S.W.K., Franklin, J., 1998. Symbolic connectionism in natural language disambiguation. IEEE Trans. Neural Netw. 9, 739–755. https://doi.org/10.1109/72.712149

Chun, M.Y., Jang, H., Kim, S.-J., Park, Y.H., Yun, J., Lockhart, S.N., Weiner, M., Carli, C.D., Moon, S.H., Choi, J.Y., Nam, K.R., Byun, B.-H., Lim, S.-M., Kim, J.P., Choe, Y.S., Kim, Y.J., Na, D.L., Kim, H.J., Seo, S.W., 2024. Emerging role of vascular burden in AT(N) classification in individuals with Alzheimer’s and concomitant cerebrovascular burdens. J. Neurol. Neurosurg. Psychiatry 95, 44–51. https://doi.org/10.1136/jnnp-2023-331603

Collins, M., 2002. Discriminative Training Methods for Hidden Markov Models: Theory and Experiments with Perceptron Algorithms, in: Proceedings of the 2002 Conference on Empirical Methods in Natural Language Processing (EMNLP 2002). Presented at the EMNLP 2002, Association for Computational Linguistics, pp. 1–8. https://doi.org/10.3115/1118693.1118694

Coyle-Gilchrist, I.T.S., Dick, K.M., Patterson, K., Vázquez Rodríquez, P., Wehmann, E., Wilcox, A., Lansdall, C.J., Dawson, K.E., Wiggins, J., Mead, S., Brayne, C., Rowe, J.B., 2016. Prevalence, characteristics, and survival of frontotemporal lobar degeneration syndromes. Neurology 86, 1736–1743. https://doi.org/10.1212/WNL.0000000000002638

de Arriba-Pérez, F., García-Méndez, S., González-Castaño, F.J., Costa-Montenegro, E., 2023. Automatic detection of cognitive impairment in elderly people using an entertainment chatbot with Natural Language Processing capabilities. J. Ambient Intell. Humaniz. Comput. 14, 16283–16298. https://doi.org/10.1007/s12652-022-03849-2

Demszky, D., Yang, D., Yeager, D.S., Bryan, C.J., Clapper, M., Chandhok, S., Eichstaedt, J.C., Hecht, C., Jamieson, J., Johnson, M., Jones, M., Krettek-Cobb, D., Lai, L., JonesMitchell, N., Ong, D.C., Dweck, C.S., Gross, J.J., Pennebaker, J.W., 2023. Using large language models in psychology. Nat. Rev. Psychol. 2, 688–701. https://doi.org/10.1038/s44159-023-00241-5

Devlin, J., Chang, M.-W., Lee, K., Toutanova, K., 2019. BERT: Pre-training of Deep Bidirectional Transformers for Language Understanding. https://doi.org/10.48550/arXiv.1810.04805

Dimitriou, T., Papatriantafyllou, J., Konsta, A., Kazis, D., Athanasiadis, L., Ioannidis, P., Koutsouraki, E., Tegos, T., Tsolaki, M., 2022. Assess of Combinations of Non-Pharmacological Interventions for the Reduction of Irritability in Patients with Dementia and their Caregivers: A Cross-Over RCT. Brain Sci. 12, 691. https://doi.org/10.3390/brainsci12060691

Dobbe, R., Krendl Gilbert, T., Mintz, Y., 2021. Hard choices in artificial intelligence. Artif. Intell. 300, 103555. https://doi.org/10.1016/j.artint.2021.103555

Doran, S.J., Sawyer, R.P., 2024. Risk factors in developing amyloid related imaging abnormalities (ARIA) and clinical implications. Front. Neurosci. 18.

Etzioni, R.S., Jesse Dodge, Noah A. Smith, Oren, n.d. Green AI [WWW Document]. URL https://cacm.acm.org/magazines/2020/12/248800-green-ai/fulltext?mobile=false (accessed 11.25.23).

Ferrari, R., Manzoni, C., Hardy, J., 2019. Genetics and molecular mechanisms of frontotemporal lobar degeneration: an update and future avenues. Neurobiol. Aging 78, 98–110. https://doi.org/10.1016/j.neurobiolaging.2019.02.006

Field, A., Coston, A., Gandhi, N., Chouldechova, A., Putnam-Hornstein, E., Steier, D., Tsvetkov, Y., 2023. Examining risks of racial biases in NLP tools for child protective services, in: 2023 ACM Conference on Fairness, Accountability, and Transparency. Presented at the FAccT ’23: the 2023 ACM Conference on Fairness, Accountability, and Transparency, ACM, Chicago IL USA, pp. 1479–1492. https://doi.org/10.1145/3593013.3594094

Fine, S., Singer, Y., Tishby, N., 1998. The Hierarchical Hidden Markov Model: Analysis and Applications. Mach. Learn. 32, 41–62. https://doi.org/10.1023/A:1007469218079

Floridi, L., Floridi, L., 2023. The Ethics of Artificial Intelligence: Principles, Challenges, and Opportunities. Oxford University Press, Oxford, New York.

Folstein, M.F., Folstein, S.E., McHugh, P.R., 1975. “Mini-mental state”. A practical method for grading the cognitive state of patients for the clinician. J. Psychiatr. Res. 12, 189–198. https://doi.org/10.1016/0022-3956(75)90026-6

Gabriel, I., 2020. Artificial Intelligence, Values, and Alignment. Minds Mach. 30, 411–437. https://doi.org/10.1007/s11023-020-09539-2

Gandy, S., 2023. News & views: anti-amyloid antibodies and novel emerging approaches to Alzheimer’s disease in 2023. Mol. Neurodegener. 18, 66. https://doi.org/10.1186/s13024-023-00656-x

Goanta, C., Aletras, N., Chalkidis, I., Ranchordas, S., Spanakis, G., 2023. Regulation and NLP (RegNLP): Taming Large Language Models. https://doi.org/10.48550/arXiv.2310.05553

Goldberg, Y., 2017. Neural Network Methods for Natural Language Processing, Synthesis Lectures on Human Language Technologies. Springer International Publishing, Cham. https://doi.org/10.1007/978-3-031-02165-7

Gorno-Tempini, M.L., Hillis, A.E., Weintraub, S., Kertesz, A., Mendez, M., Cappa, S.F., Ogar, J.M., Rohrer, J.D., Black, S., Boeve, B.F., Manes, F., Dronkers, N.F., Vandenberghe, R., Rascovsky, K., Patterson, K., Miller, B.L., Knopman, D.S., Hodges, J.R., Mesulam, M.M., Grossman, M., 2011. Classification of primary progressive aphasia and its variants. Neurology 76, 1006–1014. https://doi.org/10.1212/WNL.0b013e31821103e6

Hochreiter, S., Schmidhuber, J., 1997. Long Short-Term Memory. Neural Comput. 9, 1735–1780. https://doi.org/10.1162/neco.1997.9.8.1735

Honig, L.S., Barakos, J., Dhadda, S., Kanekiyo, M., Reyderman, L., Irizarry, M., Kramer, L.D., Swanson, C.J., Sabbagh, M., 2023. ARIA in patients treated with lecanemab (BAN2401) in a phase 2 study in early Alzheimer’s disease. Alzheimers Dement. Transl. Res. Clin. Interv. 9, e12377. https://doi.org/10.1002/trc2.12377

Hovy, D., Prabhumoye, S., 2021. Five sources of bias in natural language processing. Lang. Linguist. Compass 15, e12432. https://doi.org/10.1111/lnc3.12432

Hutchins, W.J., 2004. The Georgetown-IBM Experiment Demonstrated in January 1954, in: Frederking, R.E., Taylor, K.B. (Eds.), Machine Translation: From Real Users to Research, Lecture Notes in Computer Science. Springer, Berlin, Heidelberg, pp. 102–114. https://doi.org/10.1007/978-3-540-30194-3_12

Iadecola, C., Duering, M., Hachinski MD, V., Joutel, A., Pendlebury, S.T., Schneider, J.A., Dichgans, M., 2019. Vascular Cognitive Impairment and Dementia. J. Am. Coll. Cardiol. 73, 3326–3344. https://doi.org/10.1016/j.jacc.2019.04.034

Ismail, Z., Black, S.E., Camicioli, R., Chertkow, H., Herrmann, N., Laforce, R., Montero-Odasso, M., Rockwood, K., Rosa-Neto, P., Seitz, D., Sivananthan, S., Smith, E.E., Soucy, J.-P., Vedel, I., Gauthier, S., CCCDTD5 participants, 2020. Recommendations of the 5th Canadian Consensus Conference on the diagnosis and treatment of dementia. Alzheimers Dement. J. Alzheimers Assoc. 16, 1182–1195. https://doi.org/10.1002/alz.12105

Jabbari, E., Holland, N., Chelban, V., Jones, P.S., Lamb, R., Rawlinson, C., Guo, T., Costantini, A.A., Tan, M.M.X., Heslegrave, A.J., Roncaroli, F., Klein, J.C., Ansorge, O., Allinson, K.S.J., Jaunmuktane, Z., Holton, J.L., Revesz, T., Warner, T.T., Lees, A.J., Zetterberg, H., Russell, L.L., Bocchetta, M., Rohrer, J.D., Williams, N.M., Grosset, D.G., Burn, D.J., Pavese, N., Gerhard, A., Kobylecki, C., Leigh, P.N., Church, A., Hu, M.T.M., Woodside, J., Houlden, H., Rowe, J.B., Morris, H.R., 2020. Diagnosis Across the Spectrum of Progressive Supranuclear Palsy and Corticobasal Syndrome. JAMA Neurol. 77, 377–387. https://doi.org/10.1001/JAMANEUROL.2019.4347

Jack, C.R., Bennett, D.A., Blennow, K., Carrillo, M.C., Dunn, B., Haeberlein, S.B., Holtzman, D.M., Jagust, W., Jessen, F., Karlawish, J., Liu, E., Molinuevo, J.L., Montine, T., Phelps, C., Rankin, K.P., Rowe, C.C., Scheltens, P., Siemers, E., Snyder, H.M., Sperling, R., Contributors, 2018. NIA-AA Research Framework: Toward a biological definition of Alzheimer’s disease. Alzheimers Dement. J. Alzheimers Assoc. 14, 535–562. https://doi.org/10.1016/j.jalz.2018.02.018

Kane, A.E., Shin, S., Wong, A.A., Fertan, E., Faustova, N.S., Howlett, S.E., Brown, R.E., 2018. Sex Differences in Healthspan Predict Lifespan in the 3xTg-AD Mouse Model of Alzheimer’s Disease. Front. Aging Neurosci. 10, 172. https://doi.org/10.3389/fnagi.2018.00172

Kapasi, A., DeCarli, C., Schneider, J.A., 2017. Impact of multiple pathologies on the threshold for clinically overt dementia. Acta Neuropathol. (Berl.) 134, 171–186. https://doi.org/10.1007/s00401-017-1717-7

Kidd, C., Birhane, A., 2023. How AI can distort human beliefs. Science 380, 1222–1223. https://doi.org/10.1126/science.adi0248

Lane, C.A., Hardy, J., Schott, J.M., 2018. Alzheimer’s disease. Eur. J. Neurol. 25, 59–70. https://doi.org/10.1111/ene.13439

Lenat, D.B., Prakash, M., Shepherd, M., 1985. CYC: Using Common Sense Knowledge to Overcome Brittleness and Knowledge Acquisition Bottlenecks. AI Mag. 6, 65–65. https://doi.org/10.1609/aimag.v6i4.510

Livingston, G., Huntley, J., Sommerlad, A., Ames, D., Ballard, C., Banerjee, S., Brayne, C., Burns, A., Cohen-Mansfield, J., Cooper, C., Costafreda, S.G., Dias, A., Fox, N., Gitlin, L.N., Howard, R., Kales, H.C., Kivimäki, M., Larson, E.B., Ogunniyi, A., Orgeta, V., Ritchie, K., Rockwood, K., Sampson, E.L., Samus, Q., Schneider, L.S., Selbæk, G., Teri, L., Mukadam, N., 2020. Dementia prevention, intervention, and care: 2020 report of the Lancet Commission. The Lancet 396, 413–446. https://doi.org/10.1016/S0140-6736(20)30367-6

Loconte, R., Orrù, G., Tribastone, M., Pietrini, P., Sartori, G., 2023. Challenging ChatGPT ’ *Intelligence*’ with Human Tools: A Neuropsychological Investigation on Prefrontal Functioning of a Large Language Model. https://doi.org/10.2139/ssrn.4377371

Meskó, B., Topol, E.J., 2023. The imperative for regulatory oversight of large language models (or generative AI) in healthcare. Npj Digit. Med. 6, 1–6. https://doi.org/10.1038/s41746-023-00873-0

Minaee, S., Azimi, E., Abdolrashidi, A., 2019. Deep-Sentiment: Sentiment Analysis Using Ensemble of CNN and Bi-LSTM Models. https://doi.org/10.48550/arXiv.1904.04206

Mioshi, E., Dawson, K., Mitchell, J., Arnold, R., Hodges, J.R., 2006. The Addenbrooke’s Cognitive Examination Revised (ACE-R): a brief cognitive test battery for dementia screening. Int. J. Geriatr. Psychiatry 21, 1078–1085. https://doi.org/10.1002/gps.1610

Mireshghallah, F., Goyal, K., Uniyal, A., Berg-Kirkpatrick, T., Shokri, R., 2022. Quantifying Privacy Risks of Masked Language Models Using Membership Inference Attacks, in: Goldberg, Y., Kozareva, Z., Zhang, Y. (Eds.), Proceedings of the 2022 Conference on Empirical Methods in Natural Language Processing. Presented at the EMNLP 2022, Association for Computational Linguistics, Abu Dhabi, United Arab Emirates, pp. 8332–8347. https://doi.org/10.18653/v1/2022.emnlp-main.570

Murley, A.G., Coyle-Gilchrist, I., Rouse, M.A., Jones, P.S., Li, W., Wiggins, J., Lansdall, C., Rodríguez, P.V., Wilcox, A., Tsvetanov, K.A., Patterson, K., Lambon Ralph, M.A., Rowe, J.B., 2020. Redefining the multidimensional clinical phenotypes of frontotemporal lobar degeneration syndromes. Brain 143, 1555–1571. https://doi.org/10.1093/brain/awaa097

Nandi, A., Counts, N., Chen, S., Seligman, B., Tortorice, D., Vigo, D., Bloom, D.E., 2022. Global and regional projections of the economic burden of Alzheimer’s disease and related dementias from 2019 to 2050: A value of statistical life approach. eClinicalMedicine 51, 101580. https://doi.org/10.1016/j.eclinm.2022.101580

Nasreddine, Z.S., Phillips, N.A., Bédirian, V., Charbonneau, S., Whitehead, V., Collin, I., Cummings, J.L., Chertkow, H., 2005. The Montreal Cognitive Assessment, MoCA: a brief screening tool for mild cognitive impairment. J. Am. Geriatr. Soc. 53, 695–699. https://doi.org/10.1111/j.1532-5415.2005.53221.x

Nichols, E., Steinmetz, J.D., Vollset, S.E., Fukutaki, K., Chalek, J., Abd-Allah, F., Abdoli, A., Abualhasan, A., Abu-Gharbieh, E., Akram, T.T., Hamad, H.A., Alahdab, F., Alanezi, F.M., Alipour, V., Almustanyir, S., Amu, H., Ansari, I., Arabloo, J., Ashraf, T., Astell-Burt, T., Ayano, G., Ayuso-Mateos, J.L., Baig, A.A., Barnett, A., Barrow, A., Baune, B.T., Bã, Y., Bezabhe, W.M.M., Bezabih, Y.M., Bhagavathula, A.S., Bhaskar, S., Bhattacharyya, K., Bijani, A., Biswas, A., Bolla, S.R., Boloor, A., Brayne, C., Brenner, H., Burkart, K., Burns, R.A., CÃ, L.A., Cao, C., Carvalho, F., Castro-de-Araujo, L.F.S., Cerin, E., Chavan, P.P., Cherbuin, N., Chu, D.-T., Costa, V.M., Couto, R.A.S., Dadras, O., Dai, X., Dandona, L., Dandona, R., Cruz-GÃ, V.D. la, Dhamnetiya, D., Silva, D.D. da, Diaz, D., Douiri, A., Edvardsson, D., Ekholuenetale, M., Sayed, I.E., El-Jaafary, S.I., Eskandari, K., Eskandarieh, S., Esmaeilnejad, S., Fares, J., Faro, A., Farooque, U., Feigin, V.L., Feng, X., Fereshtehnejad, S.-M., Fernandes, E., Ferrara, P., Filip, I., Fillit, H., Fischer, F., Gaidhane, S., Galluzzo, L., Ghashghaee, A., Ghith, N., Gialluisi, A., Gilani, S.A., Glavan, I.-R., Gnedovskaya, E.V., Golechha, M., Gupta, R., Gupta, V.B., Gupta, V.K., Haider, M.R., Hall, B.J., Hamidi, S., Hanif, A., Hankey, G.J., Haque, S., Hartono, R.K., Hasaballah, A.I., Hasan, M.T., Hassan, A., Hay, S.I., Hayat, K., Hegazy, M.I., Heidari, G., Heidari-Soureshjani, R., Herteliu, C., Househ, M., Hussain, R., Hwang, B.-F., Iacoviello, L., Iavicoli, I., Ilesanmi, O.S., Ilic, I.M., Ilic, M.D., Irvani, S.S.N., Iso, H., Iwagami, M., Jabbarinejad, R., Jacob, L., Jain, V., Jayapal, S.K., Jayawardena, R., Jha, R.P., Jonas, J.B., Joseph, N., Kalani, R., Kandel, A., Kandel, H., Kasa, A.S., Kassie, G.M., Keshavarz, P., Khan, M.A., Khatib, M.N., Khoja, T.A.M., Khubchandani, J., Kim, M.S., Kim, Y.J., Kisa, A., Kisa, S., KivimÃ, M., Koroshetz, W.J., Koyanagi, A., Kumar, G.A., Kumar, M., Lak, H.M., Leonardi, M., Li, B., Lim, S.S., Liu, X., Liu, Y., Logroscino, G., Lorkowski, S., Lucchetti, G., Saute, R.L., Magnani, F.G., Malik, A.A., Mehndiratta, M.M., Menezes, R.G., Meretoja, A., Mohajer, B., Ibrahim, N.M., Mohammad, Y., Mohammed, A., Mokdad, A.H., Mondello, S., Moni, M.A.A., Moniruzzaman, M., Mossie, T.B., Nagel, G., Naveed, M., Nayak, V.C., Kandel, S.N., Nguyen, T.H., Oancea, B., Otstavnov, N., Otstavnov, S.S., Owolabi, M.O., Panda-Jonas, S., Kan, F.P., Pasovic, M., Patel, U.K., Pathak, M., Peres, M.F.P., Perianayagam, A., Peterson, C.B., Phillips, M.R., Pinheiro, M., Piradov, M.A., Pond, C.D., Potashman, M.H., Pottoo, F.H., Prada, S.I., Radfar, A., Raggi, A., Rahim, F., Rahman, M., Ram, P., Ranasinghe, P., Rawaf, D.L., Rawaf, S., Rezaei, N., Rezapour, A., Robinson, S.R., Romoli, M., Roshandel, G., Sahathevan, R., Sahebkar, A., Sahraian, M.A., Sathian, B., Sattin, D., Sawhney, M., Saylan, M., Schiavolin, S., Seylani, A., Sha, F., Shaikh, M.A., Shaji, K.S., Shannawaz, M., Shetty, J.K., Shigematsu, M., Shin, J.I., Shiri, R., Silva, D.A.S., Silva, P., Silva, R., Singh, J.A., Skryabin, V.Y., Skryabina, A.A., Smith, A.E., Soshnikov, S., Spurlock, E.E., Stein, D.J., Sun, J., TabarÃ, R., Thakur, B., Timalsina, B., Tovani-Palone, M.R., Tran, B.X., Tsegaye, G.W., Tahbaz, S.V., Valdez, P.R., Venketasubramanian, N., Vlassov, V., Vu, G.T., Vu, L.G., Wang, Y.-P., Wimo, A., Winkler, A.S., Yadav, L., Jabbari, S.H.Y., Yamagishi, K., Yang, L., Yano, Y., Yonemoto, N., Yu, C., Yunusa, I., Zadey, S., Zastrozhin, M.S., Zastrozhina, A., Zhang, Z.-J., Murray, C.J.L., Vos, T., Collaborators, D.F., 2022. Estimation of the global prevalence of dementia in 2019 and forecasted prevalence in 2050: an analysis for the Global Burden of Disease Study 2019. Lancet Public Health 7, e105–e125. https://doi.org/10.1016/S2468-2667(21)00249-8

Norwegian Consumer Council, 2023. Ghost in the machine - addressing the consumer harms of generative AI (No. https://storage02.forbrukerradet.no/media/2023/06/generative-ai-rapport-2023.pdf).

Olney, N.T., Spina, S., Miller, B.L., 2017. Frontotemporal Dementia. Neurol. Clin. 35, 339–374. https://doi.org/10.1016/j.ncl.2017.01.008

Parikh, A.P., Täckström, O., Das, D., Uszkoreit, J., 2016. A Decomposable Attention Model for Natural Language Inference. https://doi.org/10.48550/arXiv.1606.01933

Peterson, K.A., Jones, P.S., Patel, N., Tsvetanov, K.A., Ingram, R., Cappa, S.F., Lambon Ralph, M.A., Patterson, K., Garrard, P., Rowe, J.B., 2021. Language Disorder in Progressive Supranuclear Palsy and Corticobasal Syndrome: Neural Correlates and Detection by the MLSE Screening Tool. Front. Aging Neurosci. 13, 675739. https://doi.org/10.3389/fnagi.2021.675739

Poos, J.M., Russell, L.L., Peakman, G., Bocchetta, M., Greaves, C.V., Jiskoot, L.C., van der Ende, E.L., Seelaar, H., Papma, J.M., van den Berg, E., Pijnenburg, Y.A.L., Borroni, B., Sanchez-Valle, R., Moreno, F., Laforce, R., Graff, C., Synofzik, M., Galimberti, D., Rowe, J.B., Masellis, M., Tartaglia, C., Finger, E., Vandenberghe, R., de Medonça, A., Tagliavini, F., Butler, C.R., Santana, I., Ber, I.L., Gerhard, A., Ducharme, S., Levin, J., Danek, A., Otto, M., Sorbi, S., Pasquier, F., van Swieten, J.C., Rohrer, J.D., Genetic FTD Initiative, GENF, 2021. Impairment of episodic memory in genetic frontotemporal dementia: A GENFI study. Alzheimers Dement. Amst. Neth. 13, e12185. https://doi.org/10.1002/dad2.12185

Prince, M., Wimo, A., Guerchet, A., Ali, G.-C., Wu, Y.-T., Prina, M., 2015. World Alzheimer Report 2015, The Global Impact of Dementia: An analysis of prevalence, incidence, cost and trends, Alzheimer’s Disease International. Alzheimer’s Disease International (ADI), London, UK.

Radford, A., Narasimhan, K., Salimans, T., Sutskever, I., 2018. Improving Language Understanding by Generative Pre-Training. preprint 12.

Rascovsky, K., Hodges, J.R., Kipps, C.M., Johnson, J.K., Seeley, W.W., Mendez, M.F., Knopman, D., Kertesz, A., Mesulam, M., Salmon, D.P., Galasko, D., Chow, T.W., Decarli, C., Hillis, A., Josephs, K., Kramer, J.H., Weintraub, S., Grossman, M., Gorno-Tempini, M.-L., Miller, B.M., 2007. Diagnostic criteria for the behavioral variant of frontotemporal dementia (bvFTD): current limitations and future directions. Alzheimer Dis. Assoc. Disord. 21, S14-18. https://doi.org/10.1097/WAD.0b013e31815c3445

Rillig, M.C., Ågerstrand, M., Bi, M., Gould, K.A., Sauerland, U., 2023. Risks and Benefits of Large Language Models for the Environment. Environ. Sci. Technol. 57, 3464–3466. https://doi.org/10.1021/acs.est.3c01106

Rowe, J.B., Holland, N., Rittman, T., 2021. Progressive supranuclear palsy: diagnosis and management. Pract. Neurol. 21, 376–383. https://doi.org/10.1136/practneurol-2020-002794

Schneider, J.A., Arvanitakis, Z., Bang, W., Bennett, D.A., 2007. Mixed brain pathologies account for most dementia cases in community-dwelling older persons. Neurology 69, 2197–2204. https://doi.org/10.1212/01.wnl.0000271090.28148.24

Sheehan, B., 2012. Assessment scales in dementia. Ther. Adv. Neurol. Disord. 5, 349–358. https://doi.org/10.1177/1756285612455733

Strubell, E., Ganesh, A., McCallum, A., 2019. Energy and Policy Considerations for Deep Learning in NLP, in: Korhonen, A., Traum, D., Màrquez, L. (Eds.), Proceedings of the 57th Annual Meeting of the Association for Computational Linguistics. Presented at the ACL 2019, Association for Computational Linguistics, Florence, Italy, pp. 3645–3650. https://doi.org/10.18653/v1/P19-1355

Talat, Z., Névéol, A., Biderman, S., Clinciu, M., Dey, M., Longpre, S., Luccioni, S., Masoud, M., Mitchell, M., Radev, D., Sharma, S., Subramonian, A., Tae, J., Tan, S., Tunuguntla, D., Van Der Wal, O., 2022. You reap what you sow: On the Challenges of Bias Evaluation Under Multilingual Settings, in: Fan, A., Ilic, S., Wolf, T., Gallé, M. (Eds.), Proceedings of BigScience Episode #5 – Workshop on Challenges & Perspectives in Creating Large Language Models. Presented at the BigScience 2022, Association for Computational Linguistics, virtual+Dublin, pp. 26–41. https://doi.org/10.18653/v1/2022.bigscience-1.3

Tian, S., Jin, Q., Yeganova, L., Lai, P.-T., Zhu, Q., Chen, X., Yang, Y., Chen, Q., Kim, W., Comeau, D.C., Islamaj, R., Kapoor, A., Gao, X., Lu, Z., 2024. Opportunities and challenges for ChatGPT and large language models in biomedicine and health. Brief. Bioinform. 25, bbad493. https://doi.org/10.1093/bib/bbad493

Tsarapatsanis, D., Aletras, N., 2021. On the Ethical Limits of Natural Language Processing on Legal Text, in: Zong, C., Xia, F., Li, W., Navigli, R. (Eds.), Findings of the Association for Computational Linguistics: ACL-IJCNLP 2021. Presented at the Findings 2021, Association for Computational Linguistics, Online, pp. 3590–3599. https://doi.org/10.18653/v1/2021.findings-acl.314

Vaswani, A., Shazeer, N., Parmar, N., Uszkoreit, J., Jones, L., Gomez, A.N., Kaiser, Ł., Polosukhin, I., 2017. Attention is All you Need, in: Advances in Neural Information Processing Systems. Curran Associates, Inc., pp. 5998–6008.

Wang, Z., Li, R., Dong, B., Wang, Jie, Li, X., Liu, N., Mao, C., Zhang, W., Dong, L., Gao, J., Wang, Jianyong, 2023. Can LLMs like GPT-4 outperform traditional AI tools in dementia diagnosis? Maybe, but not today. https://doi.org/10.48550/arXiv.2306.01499

Wear, H.J., Wedderburn, C.J., Mioshi, E., Williams-Gray, C.H., Mason, S.L., Barker, R.A., Hodges, J.R., 2008. The Cambridge Behavioural Inventory revised. Dement. Neuropsychol. 2, 102–107. https://doi.org/10.1590/S1980-57642009DN20200005

Wolfe, R., Yang, Y., Howe, B., Caliskan, A., 2023. Contrastive Language-Vision AI Models Pretrained on Web-Scraped Multimodal Data Exhibit Sexual Objectification Bias, in: 2023 ACM Conference on Fairness, Accountability, and Transparency. pp. 1174–1185. https://doi.org/10.1145/3593013.3594072

Wu, Y., Schuster, M., Chen, Z., Le, Q.V., Norouzi, M., Macherey, W., Krikun, M., Cao, Y., Gao, Q., Macherey, K., Klingner, J., Shah, A., Johnson, M., Liu, X., Kaiser, Ł., Gouws, S., Kato, Y., Kudo, T., Kazawa, H., Stevens, K., Kurian, G., Patil, N., Wang, W., Young, C., Smith, J., Riesa, J., Rudnick, A., Vinyals, O., Corrado, G., Hughes, M., Dean, J., 2016. Google’s Neural Machine Translation System: Bridging the Gap between Human and Machine Translation. CoRR abs/1609.08144.

Zucchella, C., Sinforiani, E., Tamburin, S., Federico, A., Mantovani, E., Bernini, S., Casale, R., Bartolo, M., 2018. The Multidisciplinary Approach to Alzheimer’s Disease and Dementia. A Narrative Review of Non-Pharmacological Treatment. Front. Neurol. 9. https://doi.org/10.3389/fneur.2018.01058
